# Supplementary material for: UbiREAD deciphers proteasomal degradation code of homotypic and branched K48 and K63 ubiquitin chains
Source: Mol Cell. Author manuscript; Available in PMC 2025 Jun 18. (PMC7617769; doi:10.1016/j.molcel.2025.02.021)
Supplement: Supplementary file 1 [file EMS206380-supplement-Supplementary_file_1.pdf]

Molecular Cell, Volume *85*

Supplemental information

UbiREAD deciphers proteasomal degradation code of homotypic and branched K48 and K63 ubiquitin chains

Leo Kiss, Leo C. James, and Brenda A. Schulman

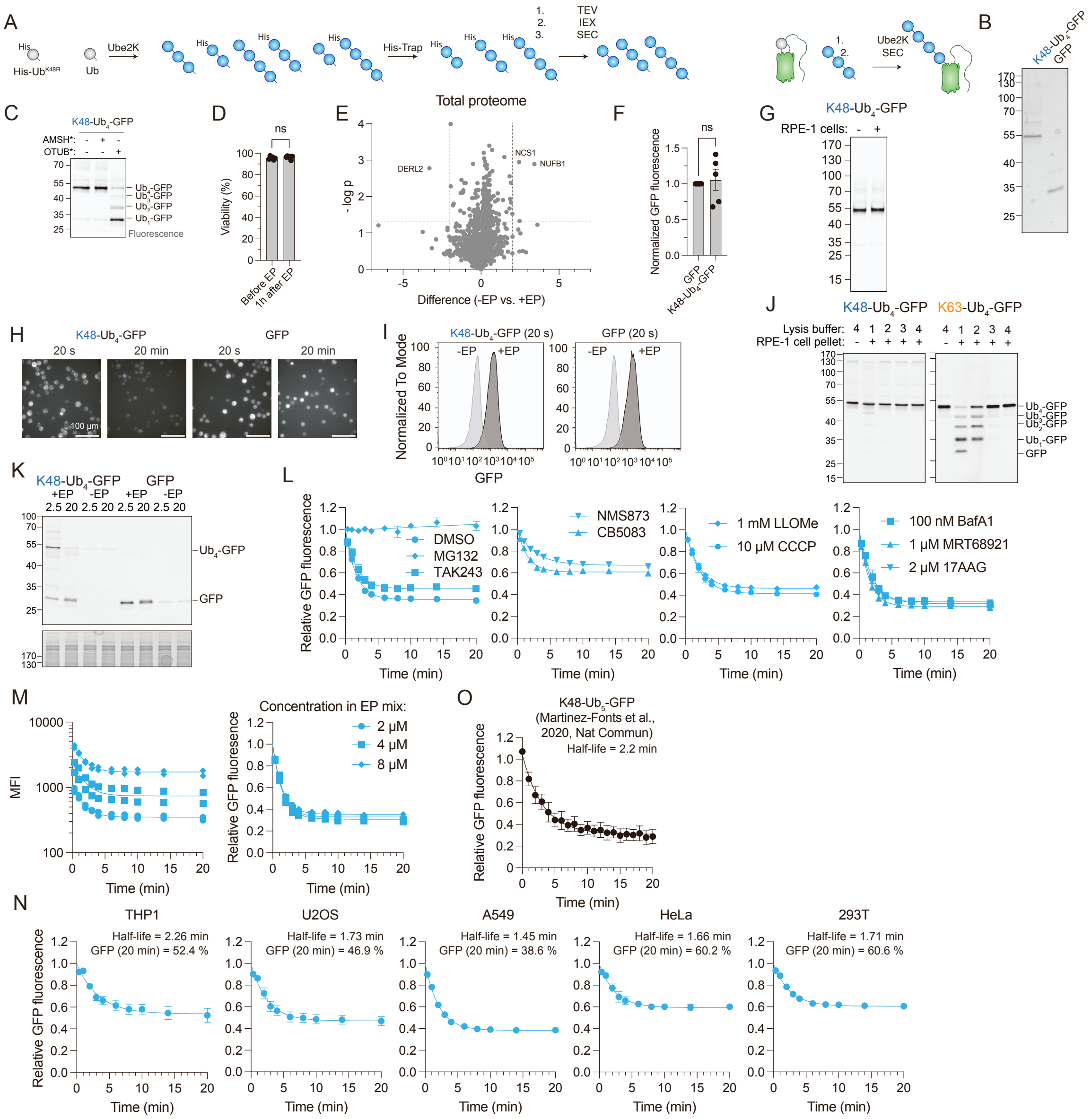

Figure S1. UbiREAD surveys intracellular ubiquitin-dependent degradation, Related to Figure 1

A Synthesis and purification strategy for capped Ub chains and their conjugation to Ub-POI.[S1] His-tagged Ub-K48R (same for K63 homotypic chains prepared in Figure 3) was incubated with Ub, E1 and E2 enzyme to generate homotypic Ub chains, where the distal Ub cannot be elongated any further. Capped and uncapped Ub chains are separated using the His-tag on the capped chains. After His-Tag cleavage of capped chains these are separated by length using cation exchange chromatography (IEX) and by size exclusion chromatography (SEC). Capped Ub chains are conjugated onto Ub-GFP using the same linkage specific E2 enzyme that was used to form the chains. Ub_n-GFP and Ub-GFP are separated by SEC.

B Coomassie stain of gel shown in Figure 1B of GFP and K48-Ub_4-GFP. Of note, additional lighter bands correspond to unfolded Ub_n-GFP.

C UbiCRest of K48-Ub_4-GFP.

D Viability determined by Trypan Blue exclusion assay before and 1 h after electroporation. Statistics originate from unpaired parametric T test (two-tailed), p=0.3343 (ns).

E Total proteome of RPE-1 cells 1 h after they were either not electroporated (-EP) or electroporated (+EP).

F K48-Ub_4-GFP delivery normalized to GFP delivery (20 s after electroporation). Statistics originate from unpaired parametric T test (two-tailed), p=0.7264 (ns).

G K48-Ub_4-GFP was incubated with RPE-1 cells exactly as in regular electroporation experiment. After 30 s, cells were not electroporated but centrifuged down and the supernatant was added to SDS buffer and run on SDS PAGE to observe Ub_n-GFP stability during cell incubation.

H Widefield microscopy showing GFP fluorescence of A549 cells.

I Flow Cytometry data testing background of non-delivered protein. K48-Ub_4-GFP or GFP were either electroporated or not electroporated and regular protocol for experiment was followed. Data show that without electroporation, no signal can be observed showing that detected GFP fluorescence is intracellular.

J Lysis buffer optimization for in gel fluorescence experiments. Non electroporated RPE-1 cell pellet was incubated with K48- or K63-Ub_4-GFP and lysis was performed in 1x RIPA buffer + c0mplete protease inhibitors (1), + 10 mM N-Ethyl-Maleimide (NEM, 2), + 100 mM NEM (3) or + 100 mM NEM + 5 mM EDTA (4). Lysis buffer 3 was chosen.

K In gel fluorescence of K48-Ub_4-GFP or GFP that were either electroporated (EP) or not in presence of RPE-1 cells to test that measured signal is intracellular rather than extracellular.

L GFP signal over time of K48-Ub_4-GFP in presence of 0.1 % DMSO, 1 μM TAK243, 5 μM MG132, 1 mM LLOMe, 10 μM CCCP, 100 nM BafA1, 1 μM MRT68921, 2 μM 17AAG, 10 μM CB5083 or 5 μM NMS873.

M Relative GFP signal over time as in Figure 1G, but here the amount K48-Ub_4-GFP was titrated and data is shown both as MFI (median fluorescence intensity) or relative GFP fluorescence. Data of the two biological replicates are shown individually.

N Relative GFP signal over time of K48-Ub_4-GFP in THP-1, U2OS, A549, HeLa and 293T cells. Error bars represent n independent experiments with n = 4 (THP-1, U2OS) or 3 (A549, HeLa, 293T).

O Biochemical single turnover assay of K48-Ub_5-GFP by yeast 26S proteasomes. Data from Source data of Martinez-Fonts et al.[S2] and fit to single exponential decay function as described for UbiREAD in methods.

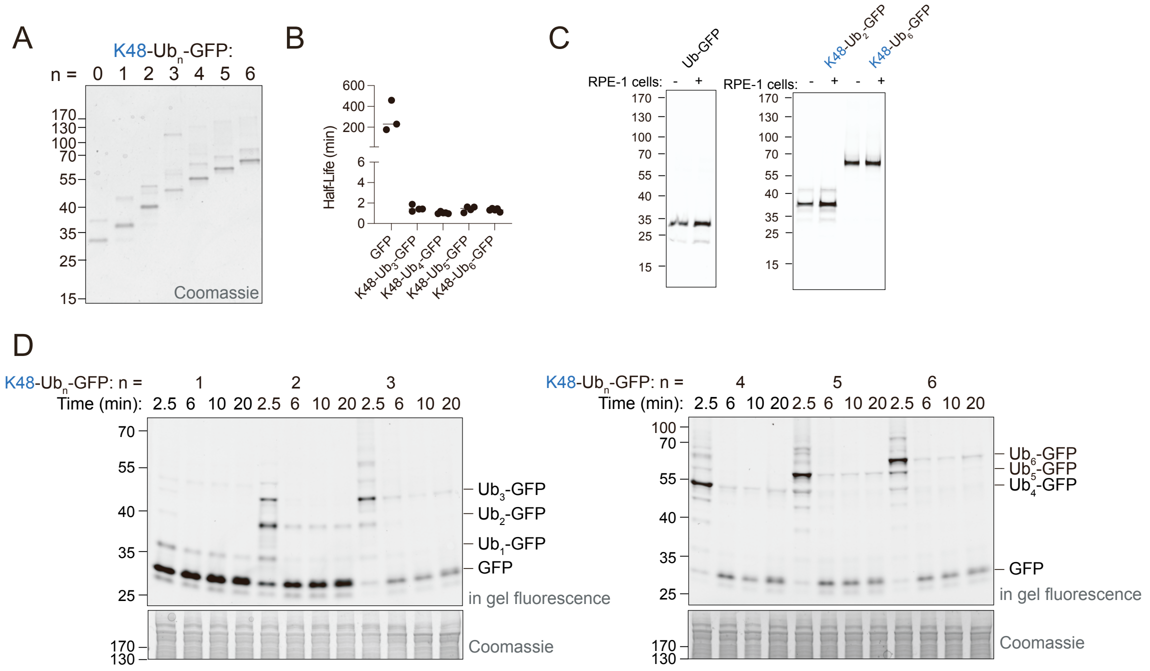

Figure S2. K48-Ub$_3$ is the minimal intracellular degradation signal, Related to Figure 2

A Coomassie stain of gel shown in Figure 2A of non-boiled K48-Ub$_n$-GFP. Of note, additional lighter bands correspond to unfolded Ub$_n$-GFP.

B Half-lives of K48-Ub$_{3/4/5/6}$-GFP and GFP of kinetics shown in Figure 2B.

C K48-Ub$_{2/6}$-GFP and Ub-GFP were incubated with RPE-1 cells exactly as in regular electroporation experiment. After 30 s, cells were not electroporated but centrifuged down and the supernatant was added to SDS buffer and run on SDS PAGE to observe Ub$_n$-GFP stability cell incubation.

D In gel fluorescence of K48-Ub$_n$-GFP delivered into RPE-1 cells showing data at 2.5, 6, 10, 20 min. Only the first and last timepoint are present in the gel in Figure 2D.

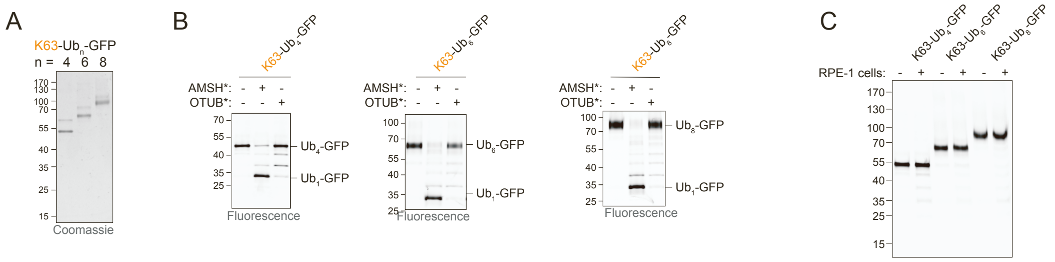

Figure S3. K63 ubiquitin chains are rapidly deubiquitinated, Related to Figure 3

A Coomassie stain of gel shown in Figure 3A of non-boiled K63-Ub_n-GFP. Of note, additional lighter bands correspond to unfolded Ub_n-GFP.

B UbiCRest of K63-$Ub_{4/6/8}$-GFP.

C K63-$Ub_{4/6/8}$-GFP were incubated with RPE-1 cells exactly as in regular electroporation experiment. After 30 s, cells were not electroporated but centrifuged down and the supernatant was added to SDS buffer and run on SDS PAGE to observe Ub_n-GFP stability during cell incubation.

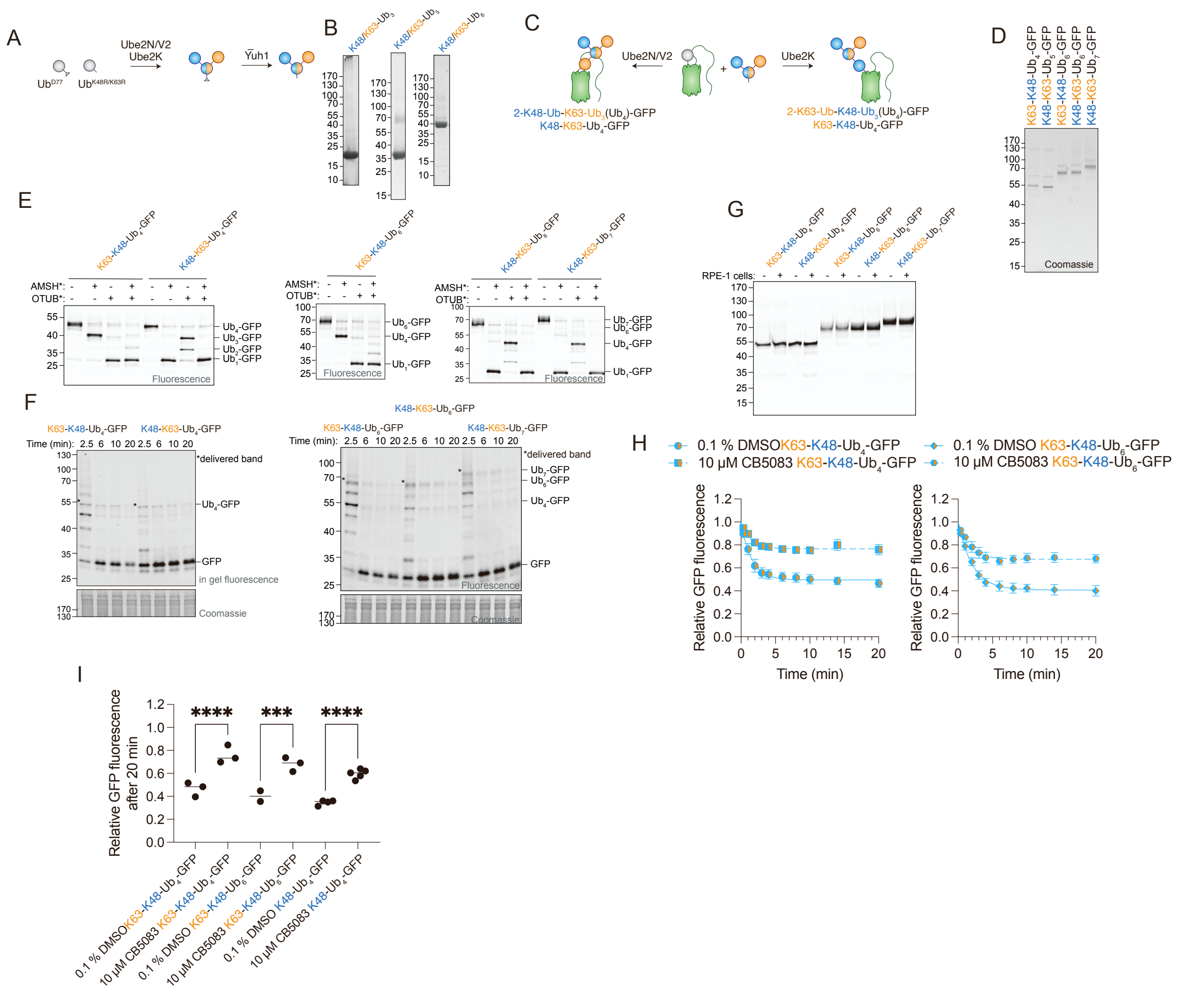

Figure S4. K48/K63-branched chains establish a degradation code inside cells, Related to Figure 4

A Synthesis strategy for K48/K63-Ub_3 (1-K48-Ub-K63-Ub_2(Ub_3)).

B Coomassie SDS PAGE gel of non-boiled K48/K63-$Ub_{3/5/6}$.

C Synthesis strategy for the conjugation of K48/K63-branched Ub_3 to Ub-GFP to generate 2-K48-Ub-K63-Ub_3(Ub_4)-GFP/K48-K63-Ub_4-GFP and K63-Ub-K48-Ub_3(Ub_4)-GFP/K63-K48-Ub_4-GFP.

D Coomassie stain of gel shown in Figure 4E of K48/K63-$Ub_{4/6/7}$-GFP. Of note, additional lighter bands correspond to unfolded Ub_n-GFP.

E UbiCRest of K48/K63-$Ub_{4/6/7}$-GFP.

F In gel fluorescence of K48/63-$Ub_{4/6/7}$-GFP delivered into RPE-1 cells. *(Asterisk) marks the delivered species.

G K48/K63-$Ub_{4/6/7}$-GFP were incubated with RPE-1 cells exactly as in regular electroporation experiment. After 30 s, cells were not electroporated but centrifuged down and the supernatant was added to SDS buffer and run on SDS PAGE to observe Ub_n-GFP stability during cell incubation.

H Relative GFP signal over time of K63-K48-$Ub_{4/6}$-GFP in presence of 0.1 % DMSO or 10 μM CB5083.

I Relative GFP fluorescence after 20 min from cellular degradation kinetics shown in H and Figure 2C and of K48-Ub_4-GFP and K63-K48-$Ub_{4/6}$-GFP. Statistics originate from ordinary one-way ANOVA, ***$p<0.0002$, ****$p<0.0001$.

Method S1. Detailed UbiREAD (Ubiquitinated Reporter Evaluation After intracellular Delivery) protocol, related to STAR Methods

UbiREAD for degradation kinetics

Electroporation was performed using the Neon® Transfection System (Thermo Fisher). Use the Neon™ Transfektions system 10 µl-Kit.

1. **General preparation:**

- Prepare 5% FACS fixative (5 % formaldehyde, 2 mM EDTA in Dulbecco's phosphate buffered saline (DPBS, 0.2 mg mL^{-1} KCl, 0.2 mg mL^{-1} KH_2PO_4, 8 mg mL^{-1} NaCl, 2.16 mg mL^{-1} Na_2HPO_4))
- Warm up regular media, antibiotic-free media, and Trypsin-EDTA mix to 37 ˚C
- Prepare 2 µL protein for electroporation in 1.5 mL sterile Eppendorf tubes
- Prepare Neon cuvette with ~3.5 mL electroporation (E) buffer
- Add 180 µL ice-cold FACS fixative into 96-well plate (or Eppendorf tube)
- Have DPBS at room temperature ready for washing cells and at 4 ˚C for changing the media of the fixed cells before flow cytometry
- Prepare 0.5 mL warm antibiotic-free media in 1.5 mL Eppendorf tubes

2. **Preparation of the cells:**

- Wash cells gently with 5 - 10 mL DPBS (RT)
- Trypsinize cells, then harvest with media and centrifuge cells (400 xg for 2 min)
- Resuspend cells in 10 mL DPBS, count and check the cellular viability (ideally >90 %)
- Take n (number of reactions + 2) x 1 x 10^6 cells and centrifuge for 2 min at 400 xg
- Aspirate supernatant and resuspend in ~1 mL DPBS and transfer cells into 1.5 mL Eppendorf tube
- Centrifuge for 2 min at 1,100 xg and aspirate supernatant
- Resuspend cells in (n + 2) x 10 µL buffer R

3. **Electroporation:**

- Add 11 µL of cells to 2 µL of protein in a 1.5 mL tube for electroporation
- Take up sample using Neon pipette and perform electroporation (1,400 V, 20 ms, 2x for RPE-1 or 1,200 V instead for 293T, U2OS, A549, THP-1)
- Add cells into 0.5 mL pre-warmed antibiotic-free media in 1.5 mL Eppendorf tube and mix
- Take out 40 µL and add into FACS fixative in ice-cold 96-well plate
- Store Eppendorf tubes with open lids in an incubator at 37 ˚C and 5 % CO_2

4. **Flow Cytometry:**

- Centrifuge 96-well plate at 600 xg for at least 5 min
- Aspirate supernatant and resuspend in 200 µL ice-cold DPBS
- Measure fluorescence at flow cytometer: use forward and side scatterer to assess live cells, then select single cells using front scatterer and peak height and measure GFP fluorescence of live single cells
- For measuring background fluorescence, either use cells that were electroporated with buffer or cells that were not electroporated (both show identical data)

5. **Data analysis**

- Export median fluorescence intensity (MFI) from data with at least 10,000 single live cells
- After background subtraction, fit data to a single exponential decay function: $Y = (Y_0 - Plateau) * e^{-k*t} + Plateau$, where Y is MFI, t is time (min), and k the rate of the function and normalize data to Y_0, to assess what happened in the dead time (20 s) of the experiment
- Estimate the half-life using $Half - life = \ln(2)/k$.

UbiREAD with in-gel fluorescence

Electroporation was performed using the Neon® Transfection System (Thermo Fisher). Use the Neon™ Transfektions system 100 µl-Kit.

Day 1:

1. **General preparation:**

- Warm up regular media, antibiotic-free media, and Trypsin-EDTA mix to 37 ˚C
- Prepare 18 µL protein for electroporation in 1.5 mL sterile Eppendorf tubes
- Prepare Neon cuvette with ~3.5 mL electroporation (E2) buffer
- Have DPBS at room temperature ready for washing cells and at 4 ˚C for changing the media of the fixed cells before flow cytometry
- Prepare ice-cold antibiotic-free media in 1.5 mL Eppendorf tubes (volume dependent on number of time-points; e.g. use 1.1 mL for 4 time-points)
- Prepare 1 mL ice-cold antibiotic-free media in 1.5 mL Eppendorf tubes for harvesting (1 / time-point)
- Prepare lysis buffer: 1x Radioimmunoprecipitation assay (RIPA) buffer (50 mM Tris-HCl pH 7.4, 150 mM NaCl, 0.25 % deoxycholic acid, 1 % NP-40, 1 mM EDTA), 1x Roche c0mplete® protease inhibitor tables, 100 mM NEM (50 µL / sample)
- Prepare 5xSDS buffer (10 g SDS, 31.2 mL Tris pH 6.8, 25 mL beta-Mercaptoethanol, 2.5 mL 2 % Bromphenol Blue, 100 mL Glycerol)
- Have a large tank of liquid nitrogen ready

2. **Preparation of the cells:**

- Wash cells gently with 5 - 10 mL DPBS (RT)
- Trypsinize cells, then harvest with media and centrifuge cells (400 xg for 2 min)
- Resuspend cells in 10 mL DPBS, count and check the cellular viability (ideally >90 %)
- Take n (number of reactions + 1) x 8 x 10^6 cells (in case of 4 time-points) and centrifuge for 2 min at 400 xg
- Aspirate supernatant and resuspend in ~1 mL DPBS and transfer cells into 1.5 mL Eppendorf tube
- Centrifuge for 2 min at 1,100 xg and aspirate supernatant
- Resuspend cells in (n + 1) x 90 µL buffer R

3. **Electroporation:**

- Add 100 µL of cells to 18 µL protein in 1.5 mL tube for electroporation
- Take up sample using Neon pipette and perform electroporation (1,400 V, 20 ms, 2x)
- Add cells into ice-cold antibiotic-free media in 1.5 mL Eppendorf tube and mix
- Take out 250 µL and add into 1 mL ice-cold media
- Centrifuge tube for 30 s at 1,100 xg, aspirate supernatant
- During centrifugation, put tube with cells into waterbath at 37 ˚C to warm them up
- Wash in 1 mL DPBS
- Centrifuge tube for 30 s at 1,100 xg, aspirate supernatant
- Put tube with cells into incubator with open lid until next harvest
- Snap-freeze pellet in liquid nitrogen
- Store pellets at -80 ˚C until further use

Day 2:

4. **Lysis and in gel fluorescence**

- Take pellets from -80 ˚C freezer and add 50 µL lysis buffer
- Perform lysis on ice for 10 min and vortex tubes every ~2 min
- Centrifuge lysates at 14,000 xg for 10 min at 4 ˚C
- Take 1 µL and add to 499 µL DPBS (for lysate concentration measurement via Micro BCA™ Protein Assay Kit)
- Take 50 µL supernatant and add to 15 µL 5x SDS buffer
- Measure lysate concentration via Micro BCA™ Protein Assay Kit according to the manufacturer description
- Load equal amounts of lysate (e.g. 20 - 40 µg) onto SDS-PAGE and run gel
- Measure in gel fluorescence on a Typhoon FLA 9500

- Afterwards, stain gel with Coomassie as loading control

5. **Data analysis**

- Band qualtification can be performed using ImageJ in FIJI
- Estimate deubiquitination rates by quantifying the deubiquitinated GFP and the main input band and fit data as described above. A value of 0 can be added for timepoint 0, since the delivered Ub_n-GFP was not deubiquitinated before delivery.

General UbiREAD Quality Control:

- Cell viability is key to a successful experiment. Check viability during counting and keep a few cells to check viability 1 h post electroporation (see data for RPE-1 cells in Figure S1D). Viability should ideally be >90 % before and after the experiment! Be particularly careful when using UbiREAD in presence of drugs as these drugs may have severe consequences for cellular health.
- Every cell line behaves differently and must be optimized and tested in electroporation. If cellular viability is low after the experiment, optimize delivery.
- Check that your protein does not get modified before electroporation for instance by incubating cells with protein of interest for the time it takes until electroporation and check the protein via SDS-PAGE (e.g. done in Figure S1G, S2C, S3C or S4G).
- Purity and homogeneity of the electroporated proteins are key for successful UbiREAD.

Supplemental References

1. Martinez-Fonts, K. and A. Matouschek, *A Rapid and Versatile Method for Generating Proteins with Defined Ubiquitin Chains.* Biochemistry, 2016. **55**(12): p. 1898-908.
2. Martinez-Fonts, K., et al., *The proteasome 19S cap and its ubiquitin receptors provide a versatile recognition platform for substrates.* Nat Commun, 2020. **11**(1): p. 477.
