# Supplementary material for: UbiREAD deciphers proteasomal degradation code of homotypic and branched K48 and K63 ubiquitin chains
Source: Mol Cell. Author manuscript; Available in PMC 2025 Jun 18. (PMC7617769; doi:10.1016/j.molcel.2025.02.021)
Supplement: Supplementary file 2 [file EMS206380-supplement-Supplementary_file_2.pdf]

Molecular Cell

Technology

UbiREAD deciphers proteasomal degradation code of homotypic and branched K48 and K63 ubiquitin chains

Graphical abstract

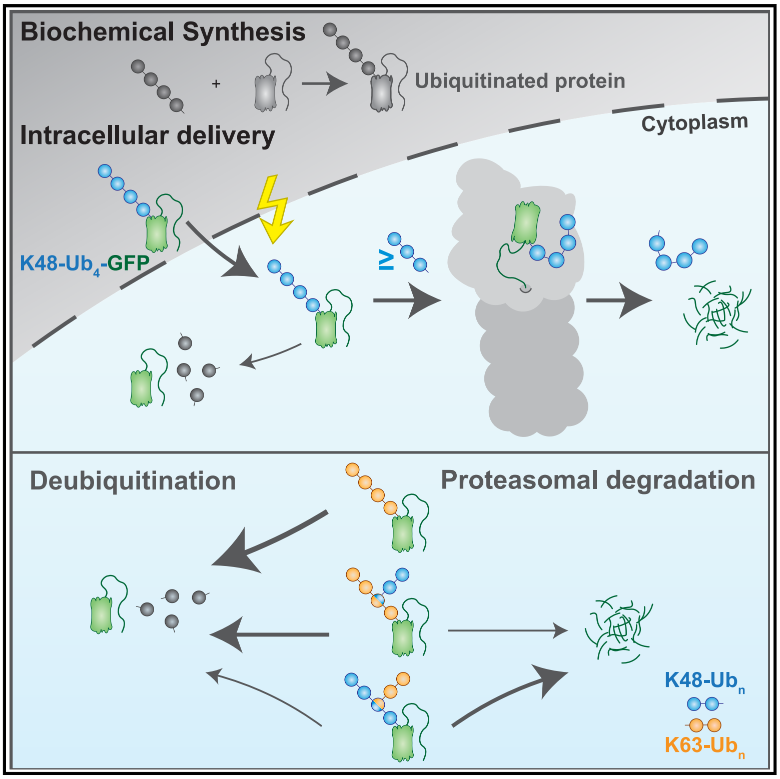

Highlights

- UbiREAD systematically compares intracellular degradation of defined substrates
- Cellular degradation occurs rapidly with a half-life of 1 min for a GFP
- K48-Ub_3 is a cellular proteasomal targeting signal
- Branched ubiquitin chains are not a sum of their parts but show a functional hierarchy

Authors

Leo Kiss, Leo C. James, Brenda A. Schulman

Correspondence

lkiss@biochem.mpg.de

In brief

Ubiquitin chains determine the fates of their modified proteins, including proteasomal degradation. Kiss et al. present UbiREAD, a technology to monitor cellular degradation and deubiquitination at high temporal resolution after intracellular delivery of ubiquitinated proteins. This reveals a degradation code for ubiquitin chains varying by linkage, length, and topology.

Kiss et al., 2025, Molecular Cell 85, 1467–1476
April 3, 2025 © 2025 The Authors. Published by Elsevier Inc.
https://doi.org/10.1016/j.molcel.2025.02.021

CellPress

Molecular Cell

CellPress
OPEN ACCESS

Technology

UbiREAD deciphers proteasomal degradation code of homotypic and branched K48 and K63 ubiquitin chains

Leo Kiss,[1,3,*] Leo C. James,[2] and Brenda A. Schulman[1]

[1]Department of Molecular Machines and Signaling, Max Planck Institute of Biochemistry, Martinsried 82152, Germany

[2]MRC Laboratory of Molecular Biology, Francis Crick Avenue, Cambridge CB2 0QH, UK

[3]Lead contact

*Correspondence: lkiss@biochem.mpg.de

https://doi.org/10.1016/j.molcel.2025.02.021

SUMMARY

Ubiquitin chains define the fates of their modified proteins, often mediating proteasomal degradation in eukaryotes. Yet heterogeneity of intracellular ubiquitination has precluded systematically comparing the degradation capacities of different ubiquitin chains. We developed ubiquitinated reporter evaluation after intracellular delivery (UbiREAD), a technology that monitors cellular degradation and deubiquitination at high temporal resolution after bespoke ubiquitinated proteins are delivered into human cells. Comparing the degradation of a model substrate modified with various K48, K63, or K48/K63-branched ubiquitin chains revealed fundamental differences in their intracellular degradation capacities. K48 chains with three or more ubiquitins triggered degradation within minutes. K63-ubiquitinated substrate was rapidly deubiquitinated rather than degraded. Surprisingly, in K48/K63-branched chains, substrate-anchored chain identity determined the degradation and deubiquitination behavior, establishing that branched chains are not the sum of their parts. UbiREAD reveals a degradation code for ubiquitin chains varying by linkage, length, and topology and a functional hierarchy within branched ubiquitin chains.

INTRODUCTION

Ubiquitin (Ub) serves as one of the most sophisticated post-translational modifications,[1,2] with most intracellular proteins undergoing ubiquitination at some point during their lifetime.[3] Ub itself is also a substrate for ubiquitination. The best-characterized linkages are between a lysine or the N terminus on one Ub and the C terminus of the next in the chain. In addition to 8 homotypic Ub chains, 10%–20% of Ub chains are branched,[4,5] meaning that a single Ub at the branch point is linked to at least two other Ubs via their C termini. The diversity of chain types was described as the Ub code,[1] wherein the Ub linkage is thought to dictate the fate of the modified substrate.

The best-recognized function of ubiquitination is eliciting 26S proteasomal degradation. However, deciphering the Ub code for degradation, one of the most fundamental biological processes, has remained a challenge. The large majority of Ub chains in cells are composed of either K48- or K63-linkages.[6] Biochemically, both K48- and K63-chains serve as degradation signals for proteasomes.[7–14] Yet, intracellular degradation is thought to rely on K48-linked Ub chains,[15–17] while K63 chains have been associated with non-degradative roles.[18] Further complexity arises from branched Ub chains. At this point, roles for such chains are unclear. Some studies have reported branched chains as superior degradation signals,[19–23] while branched chains were also reported to prevent proteasome binding.[24] However, a major challenge to understanding intrinsic differences in Ub chains is that it is not possible to disentangle the effects of chain type, length, and topology on the extent and rate of intracellular substrate degradation because ubiquitination is inherently heterogeneous.

Discrepancies between cellular and biochemical experiments underscore the need for approaches that enable the systematic comparison of Ub-mediated phenotypes inside cells. Ubiquitination of cellular proteins can be induced,[25–27] but these approaches do not overcome the challenge of heterogeneity and only enable modification of a small fraction of the targeted protein. To address this, we developed ubiquitinated reporter evaluation after intracellular delivery (UbiREAD)—a technology for the synthesis and intracellular delivery of bespoke ubiquitinated GFP that enables monitoring of cellular degradation and deubiquitination at high temporal resolution. We have used UbiREAD to uncouple ubiquitination from degradation and deubiquitination and measure the kinetics of these processes induced by different Ub-chain types inside living cells. Our data reveal that degradation of a model substrate occurs at half-lives of up to 1 min. A kinetic competition between deubiquitination and degradation is encoded in the length of the K48

This is an open access article under the CC BY license (http://creativecommons.org/licenses/by/4.0/).

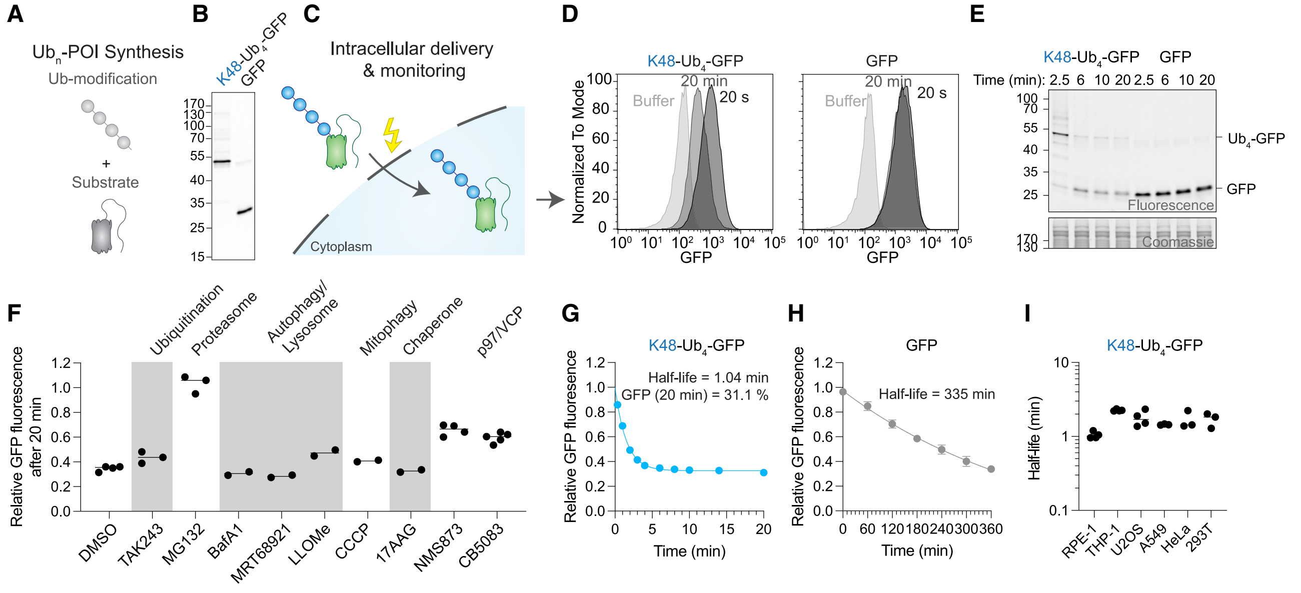

Figure 1. UbiREAD surveys intracellular ubiquitin-dependent degradation

(A and C) Cartoon describing the concept of preparing bespoke polyubiquitinated proteins *in vitro* and delivering them into the cytoplasm to measure their degradation.

(B) In-gel fluorescence of SDS-PAGE showing purity of GFP and K48-Ub$_4$-GFP. The same gel stained with Coomassie is shown in Figure S1B.

(D) Flow cytometry showing GFP fluorescence of RPE-1 cells either delivered with buffer, GFP, or K48-Ub$_4$-GFP.

(E) In-gel fluorescence of K48-Ub$_4$-GFP or GFP delivered into RPE-1 cells.

(F) Relative GFP fluorescence after 20 min in the presence of the indicated drug. Full data are shown in Figure S1L.

(G) Relative GFP signal over time of K48-Ub$_4$-GFP. Data are represented as mean ± SEM of $n = 4$ independent experiments.

(H) Relative GFP signal over time of GFP. Data are represented as mean ± SEM of $n = 3$ independent experiments.

(I) Half-lives of cellular degradation kinetics of K48-Ub$_4$-GFP in RPE-1, THP-1, U2OS, A549, HeLa, and 293T cells. Full data shown in Figure S1N.

chain: once a length of 3 Ubs is reached, degradation occurs efficiently. By contrast, K63 chains are deubiquitinated faster than K48 chains irrespective of Ub-chain length. Finally, we reveal a complex hierarchy of degradation properties encoded by K48/K63-branched Ub chains. Instead of behaving independently, the substrate-anchored chain determines whether degradation or deubiquitination occurs.

DESIGN

We sought to systematically interrogate how K48, K63, and K48/K63-branched Ub chains impact intracellular degradation of a model substrate. To achieve this, we needed defined Ub chains conjugated to a substrate, a quantifiable substrate reporter for degradation, and an intracellular delivery method. First, ubiquitinated proteins were synthesized by preparing Ub chains of defined length and composition, followed by conjugation of these to a mono-ubiquitinated GFP model degradation substrate (Figures 1A and S1A).[10] Chain length is fixed by using a distal Ub that cannot be elongated further as the lysine corresponding to the chain type is mutated to arginine (e.g., K48R for K48 chains). Second, for ease of detection, we used a GFP variant as a substrate reporter that has previously been engineered for efficient proteasomal degradation.[9,10,28] Third, our method uses electroporation for efficient cytoplasmic delivery of functional recombinant proteins (Figure 1C).[29–31] Electroporation has been demonstrated for effectiveness in interrogating the ubiquitin proteasome system (UPS).[32–35] In contrast to other protein delivery methods such as glass bead loading or cell-penetrating peptides,[36,37] electroporation occurs within ms, thereby enabling kinetic assays.

RESULTS

UbiREAD surveys intracellular Ub-dependent degradation

We synthesized Ub$_n$-GFP in mg quantities and high purity for multiple Ub-chain types (Figures 1B and S1B) and validated these by Ub-chain restriction (UbiCRest, Figure S1C).[38] Delivery of GFP into RPE-1 cells by electroporation did not show any obvious impact on cell viability or the proteome (Figures S1D and S1E). K48-Ub$_4$-GFP was delivered as efficiently as GFP and was not processed during incubation with RPE-1 cells prior to electroporation (Figures S1F and S1G). To test for intracellular degradation, we delivered either K48-Ub$_4$-GFP or GFP into RPE-1 cells and formaldehyde-fixed a fraction of the cells after 20 s or 20 min. Flow cytometry revealed that GFP fluorescence was largely lost only when the delivered GFP was conjugated with K48-Ub$_4$ (Figure 1D). Detected fluorescence was cytoplasmic and did not originate from extracellular protein (Figures S1H and S1I). We have thus established a method for preparing bespoke ubiquitinated GFP and delivering them into

cells, allowing for the systematic evaluation of Ub-chain-encoded degradation—we named this method UbiREAD.

To complement the flow cytometry reporting on GFP fluorescence, we interrogated the identity of the GFP signal during degradation by in-gel fluorescence. This approach allowed the discrimination of input and deubiquitinated species. Since harvesting the cells was not as fast as fixing, we slowed the reaction by using ice-cold buffers and found that the K48-Ub_4-GFP band was largely lost within 6 min (Figure 1E). In addition, a band at lower intensity corresponding to deubiquitinated GFP appeared within 6 min. Based on our experiments with GFP alone, we surmise that this deubiquitinated fraction is not targeted to the proteasome in the short term. Observed deubiquitination occurred inside cells, and the GFP signal represents intracellular protein (Figures S1J and S1K). The data imply a competition between degradation and deubiquitination, which in the case of K48-Ub_4-GFP, is largely won by degradation.

To further validate that UbiREAD surveys proteasomal degradation dependent on our input Ub chains, we used specific inhibitors. Indeed, targeting the proteasome using MG132 fully stabilized the substrate, while inhibition of ubiquitination using E1 inhibitor TAK243 did not reduce degradation significantly (Figures 1F and S1L). Interestingly, p97 inhibition with either CB5083 or NMS873 had an intermediate effect, while inhibition of other pathways had lesser or no effects on degradation in our system. However, effects of p97 inhibition may arise both from direct loss of p97 activity or indirect action by proteasome clogging of ubiquitinated p97 clients. Collectively, the data confirmed that in UbiREAD, the observed K48-Ub_4-GFP degradation was dependent on proteasome activity and the preassembled Ub chain rather than intracellular ubiquitination.

Intracellular K48-dependent degradation occurs rapidly

Next, we used UbiREAD to define intracellular degradation kinetics of K48-Ub_4-GFP. After 6 min, degradation had plateaued, with $\sim$30% GFP signal remaining (Figure 1G), consistent with deubiquitination. When modified with a K48-Ub_4 chain, GFP degradation occurred with a half-life of 1 min. By contrast, GFP on its own was turned over more than two orders of magnitude slower (Figure 1H). Increasing the amount of electroporated K48-Ub_4-GFP did not change the degradation half-life nor the amount of degraded substrate, indicating that the maximal intracellular degradation capacity was not reached in our experiments (Figure S1M). To test the general applicability of our approach, we also recorded degradation kinetics in THP-1, U2OS, A549, HeLa, and 293T cells (Figures 1I and S1N), which showed half-lives from 1 to 2.2 min. Thus, UbiREAD allows monitoring of intracellular degradation kinetics in diverse mammalian cell lines.

The rapid degradation rates observed here are consistent with the major role of the UPS in regulating proteostasis and are striking for two reasons. First, intracellular K48-Ub_4-GFP degradation is about twice as fast as K48-Ub_5-GFP degradation under single turnover conditions by purified yeast proteasomes (Figure S1O).[9] Notably, *in vitro* degradation kinetics of unfolded proteins are significantly faster than stably folded ones.[11,39] More rapid cellular degradation of proteins that are rate-limited by unfolding may be enabled by pre-processing via the unfoldase p97/VCP. Indeed, unfolding by purified yeast p97 (Cdc48) of K48-Ub_n-mEos (similar in size to GFP) occurred within seconds.[40] However, *in vitro* degradation kinetics measured in biochemical reconstitutions of p97 and proteasomes differ from those we measured inside cells.[40,41] Thus, the faster intracellular degradation velocities observed here are likely explained by the close collaboration of multiple molecular machines operating *in situ*. Second, when considering the rate of intracellular translation[42,43] of a protein around the size of GFP, the estimated synthesis doubling time of 0.6 to 1.7 min is on the same order as the intracellular degradation half-life we measured. Therefore, the rate of degradation by the UPS has the potential to balance protein synthesis by operating on a similar time scale.

K48-Ub_3 is the minimal intracellular proteasomal degradation signal

Since intracellularly formed Ub chains are heterogeneous in length, it is unclear how long a K48 chain must be to induce degradation. To systematically interrogate the influence of chain length on degradation, we synthesized K48-Ub_n-GFP with lengths ranging from 2 to 6 (Figures 2A and S2A) and monitored their stability in RPE-1 cells (Figure 2B). Degradation occurred rapidly and efficiently once a Ub-chain length of 3 was reached (Figures 2B, 2C, and S2B). Increasing the chain length further did not significantly accelerate degradation. We next asked whether the absence of degradation correlates with deubiquitination. Indeed, we found low deubiquitination levels of GFP carrying 3 or more Ubs but substantial deubiquitination below that (Figures 2D, S2C, and S2D). We determined K48-Ub_2-GFP deubiquitination to occur with a half-life of $\sim$2 min (Figure 2E). Deubiquitination likely occurs even faster, as the reaction was slowed by harvesting cells in ice-cold buffer. Collectively, our data identify K48-Ub_3 as the minimum intracellular proteasomal degradation motif.

We were surprised to identify K48-Ub_3 as the minimum intracellular degradation signal of our model reporter since biochemical reconstitution using yeast proteasomes had suggested that longer chains are required.[7] But inside cells, additional factors participate in degradation. In yeast, it has been suggested that most K48-modified proteins interact with Cdc48 and are delivered to proteasomes via its shuttles, including Rad23.[44,45] While both purified yeast and human p97 were shown to prefer K48 chains of 5 or longer,[40,46–48] a structure of Cdc48 and its adaptors in the process of unfolding a K48-modified substrate only resolved 3 Ubs.[49] Similarly, purified proteasome shuttle Rad23 shows maximal affinity to longer K48 chains but can already bind K48-Ub_3.[44,50] Importantly, K48 chains in yeast were found to largely comprise 3 to 5 Ubs,[51] which corresponds to the optimal length regime required for efficient degradation in human cells.

K63 Ub chains are rapidly deubiquitinated

K63 is the second most abundant Ub-chain type in cells and is mostly associated with non-proteolytic functions.[1,6,52] Nonetheless, purified yeast proteasomes degrade K63- nearly as efficiently as K48-modified substrates.[7–14] To compare the effects of K63 chains with those of K48 chains on degradation inside cells using our system, we synthesized K63-$Ub_{4/6/8}$-GFP

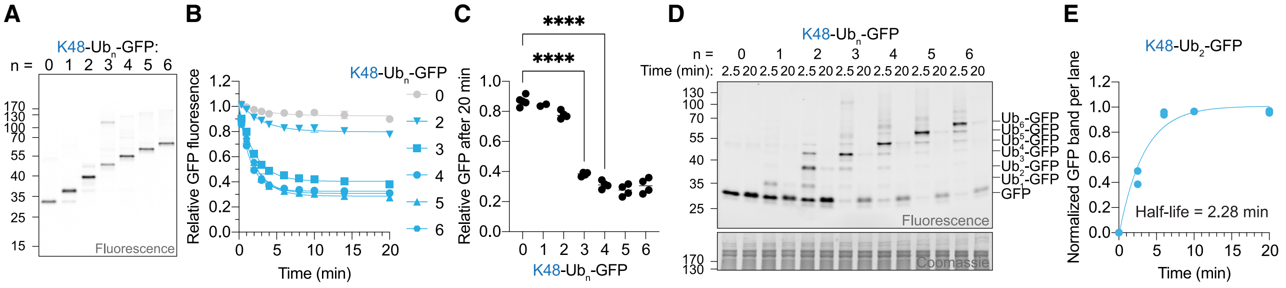

Figure 2. K48-Ub$_3$ is the minimal intracellular proteasomal degradation signal

(A) In-gel fluorescence of SDS-PAGE of non-boiled K48-Ub$_n$-GFP. Same gel with Coomassie stain is shown in Figure S2A.

(B) Relative GFP signal over time of K48-Ub$_n$-GFP in RPE-1 cells. Data are represented as mean ± SEM of n independent experiments with n = 2 (Ub-GFP), 4 (Ub$_{2/3/4/5/6}$-GFP), and 5 (GFP).

(C) Relative GFP fluorescence after 20 min from cellular degradation kinetics shown in (B). Statistics originate from ordinary one-way ANOVA, ****$p < 0.0001$.

(D) In-gel fluorescence of K48-Ub$_n$-GFP delivered into RPE-1 cells. Of note, Ub$_n$-GFP substrates can smear and show additional bands when being run on SDS-PAGE.

(E) Quantification of K48-Ub$_2$-GFP deubiquitination. Shown is the relative GFP band per lane. Data of the two biological replicates are shown individually. See also Figure S2D.

(Figures 3A, S3A, and S3B) and delivered them into RPE-1 cells to monitor their stability. However, we did not observe significant loss of fluorescence for K63 chain-conjugated GFP (Figure 3B). Since GFP remained stable, we next tested Ub-chain stability by in-gel fluorescence. We found that K63 chains were rapidly deubiquitinated, with half-lives in the 1 min range (Figures 3C–3E and S3C), which is faster than previously estimated.[53] Thus, deubiquitination may occur more rapidly than K63 chains promote entry into proteasomal degradation pathways, or K63 chains may simply lack the information required to serve as proteasomal substrates inside cells.

However, K63 chains have been observed inside cells and associated with various signaling functions. One notable explanation could be that UbiREAD exclusively monitors the fate of pre-formed chains, whereas most cellular experiments are performed during the ongoing production of new chains. Moreover, we speculate that those K63 chains that are observed in cells may be protected from deubiquitination by rapid interaction with downstream partners that might be localized nearby. Phase separation mediated by multivalent interactions of K63 chains could also protect such modifications when needed,[54–57] possibly if multiple K63 chains on a substrate would be required to drive their function. In addition, many K63-dependent functions, such as DNA damage response, are localized to the nucleus, while UbiREAD monitors cytoplasmic events. Yeast K63 linkages were largely found to be part of Ub$_2$.[51] Importantly, our data are consistent with the notion that K63 chains could be highly transient signals that are generally subject to tight regulation by the interplay between their interaction partners protecting them and their disassembly by deubiquitinases (DUBs).

Degradation hierarchy encoded within K48/K63-branched chains

It has been estimated that in human cells, 10% to 20% of Ub chains are branched,[4,5] and 20% of K63-linked Ub was suggested to be part of K48/K63 branches.[58] K48/K63-branched Ub chains have been identified to be involved in both degradation[21,22] and non-proteolytic pathways.[58,59] To interrogate their

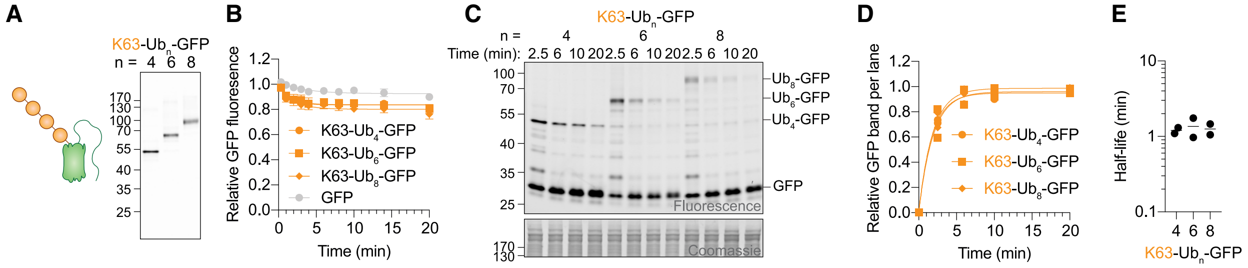

Figure 3. K63 Ub chains are rapidly deubiquitinated

(A) Cartoon schematic and in-gel fluorescence of SDS-PAGE of non-boiled K63-Ub$_n$-GFP. Same gel with Coomassie stain is shown in Figure S3A.

(B) Relative GFP signal over time of K63-Ub$_n$-GFP in RPE-1 cells. Data are represented as mean ± SEM of n independent experiments with n = 3 (Ub$_{4/8}$-GFP), 5 (GFP), and 6 (Ub$_6$-GFP).

(C) In-gel fluorescence of K63-Ub$_n$-GFP delivered into RPE-1 cells.

(D) Quantification of K63-Ub$_n$-GFP deubiquitination. Shown is the relative GFP band per lane. Data of the two biological replicates are shown individually.

(E) Deubiquitination half-lives determined from fits shown in (D).

degradation capacity, we first needed a synthesis strategy that enabled the production of GFP-carrying branched Ub chains. Initially, we generated branched Ub_3 by using $Ub^{K48R/K63R}$ together with C-terminally protected Ub^{D77} (Figure S4A). C-terminal deprotection by Yuh1 opens the formed chain up to additional conjugation reactions. To generate longer branched chains, we extended this approach by biochemically synthesizing C-terminally protected Ub_2s, followed by deprotection and subsequent rounds of synthesis and deprotection (Figures 4A and 4B). Using this strategy, we were able to generate branched K48/K63-Ub_3, -Ub_5, and -Ub_6 in mg quantities and high purity (Figure S4B). We conjugated these chains to Ub-GFP either via K48- or K63-ligation, resulting in branched Ub_4-, Ub_6-, and Ub_7-GFPs (Figures 4C–4E and S4C–S4E). Our synthesis strategy therefore enables the generation of complex K48/K63-branched Ub-chain architectures to allow their specific activities to be characterized inside cells. To describe our GFPs carrying complex branched Ub-chain topologies, we used the Kulathu lab nomenclature[60] due to its clarity for longer and more complex branched Ub chains. Importantly, K48/K63 describes all branches, including the two chain types, irrespective of topology, while K48-K63 describes chains where K63 is the main and K48 the branched chain and vice versa for K63-K48.

Having synthesized K48/K63-ubiquitinated GFPs, we next delivered them into RPE-1 cells. Interestingly, if the main chain was K63, the extent of degradation observed was low (Figures 4F and 4G). Even in the presence of a K48-Ub_3-branch from a K63-chain (K48-K63-Ub_6-GFP), we saw little indication of efficient degradation in our system. In this substrate, the proximal Ub of K48-Ub_3 is also part of the K63 chain. Therefore, we synthesized a substrate that contains K48-Ub_4 as a branched chain (Figures 4B, 4D, and 4E) to ensure the presence of a structurally discrete K48 chain with at least 3 Ubs that are only joined via a K48-linkage. However, extending the K48-branched chain did not significantly improve degradation for the GFP that was directly marked with a K63 chain (Figures 4F and 4G). GFPs carrying K63 main chains were largely deubiquitinated at rates similar to homotypic K63 chains (Figures S4F and S4G), indicating that K63-encoded DUB recruitment outcompetes K48-encoded degradation. K48-K63 branched chains have been described as degradative signals in the case of TXNIP and cIAP1[21,22] but not in the case of TRAF6.[58] Given that K48-K63 chains do not efficiently induce GFP degradation in our system, this suggests that cellular degradation of these substrates marked by K48-K63 branched chains is induced by other Ub signals.

Importantly, GFP substrates directly modified by K48 main chains and carrying K63 chains as branches (K63-K48) showed robust degradation. We directly compared these with their closest relatives carrying homotypic, unbranched K48 chains. This revealed that appending K63 branches onto K48 chains modestly reduced degradation efficiency (Figure 4G), coincident with increased deubiquitination (Figure S4F). Overall, forming K63 chains as branches on K48 chains does not improve GFP degradation. Indeed, K48/K63-branched chains were identified at sites of DNA damage and accumulated under p97 but not proteasome inhibition.[59] In addition, factors associated with DNA damage were shown to specifically recognize K48/K63 branch points.[59,61] We find the effects of p97 inhibition on K63-K48-Ub_n-GFP comparable to those on K48-Ub_4-GFP (Figures S4H and S4I), indicating that p97-specific interactions with branched chains may occur in non-proteasomal pathways or p97-specific involvement may be more important in the nucleus. We establish that in K48/K63-branched chains, the Ub chain that is substrate-anchored has priority over a secondary, distal chain. Distinct properties encoded in a proximal Ub chain (e.g., degradation by K48-Ub_3) can therefore be lost within the context of a distal chain. This functional hierarchy adds another layer of complexity to the Ub code.

DISCUSSION

We have developed UbiREAD for preparing and delivering GFP modified with distinct Ub-chain types, lengths, and topologies into cells. Notably, UbiREAD enables the systematic comparison of Ub-chain-encoded degradation and deubiquitination in the cytoplasmic milieu at high temporal resolution. We found that degradation induced by K48-ubiquitination occurs as rapidly as ribosomal translation. In addition, a kinetic competition between deubiquitination and degradation is regulated by the length of the K48 chain, with Ub_3 turning on degradation. By contrast, K63 chains never induced efficient degradation irrespective of chain length and were instead disassembled with rates that were even faster than for K48 chains. Surprisingly, K48/K63-branched Ub chains showed distinct phenotypes depending on the order of assembly, establishing that branched Ub chains behave co-dependently. UbiREAD therefore reveals a degradation code for Ub chains varying by linkage, length, and topology, thereby shedding light on one of the most fundamental processes inside cells.

Intracellular degradation of K48 ubiquitinated GFP was largely consistent with biochemical reconstitution assays but with several key differences. Degradation rates inside cells were faster compared with single turnover kinetics using yeast proteasomes[9] (Figure 1), and a shorter Ub chain than identified *in vitro*[7] was sufficient to induce efficient intracellular degradation (Figure 2). Differences between biochemical and intracellular data were more pronounced for K63 chains, which were shown to induce efficient degradation by purified yeast proteasomes,[7–14] but were rapidly deubiquitinated inside cells (Figure 3). These findings highlight the importance of investigating Ub chains and their functions in their native cellular environment.

Our work also establishes a readily adaptable platform for analyzing intracellular degradation of model substrates conjugated with pre-formed Ub and Ub-like protein (Ubl) chains. UbiREAD is thus also applicable to the investigation of intracellular degradation and deubiquitination capacities of Ub chains other than K48 or K63, which have also been implicated in degradation. In addition, Ub chains may also be combined with other post-translational modifications, such as phosphorylation or acetylation. While we have used a model degradation substrate, this could be readily replaced with more challenging substrates that are predicted to be degraded more slowly and less efficiently.

While K48 and K63 linkages are predominant cellular Ub signals, other linkage types are also present,[6] including in branched chains. For instance, K11/K48 chains are formed by

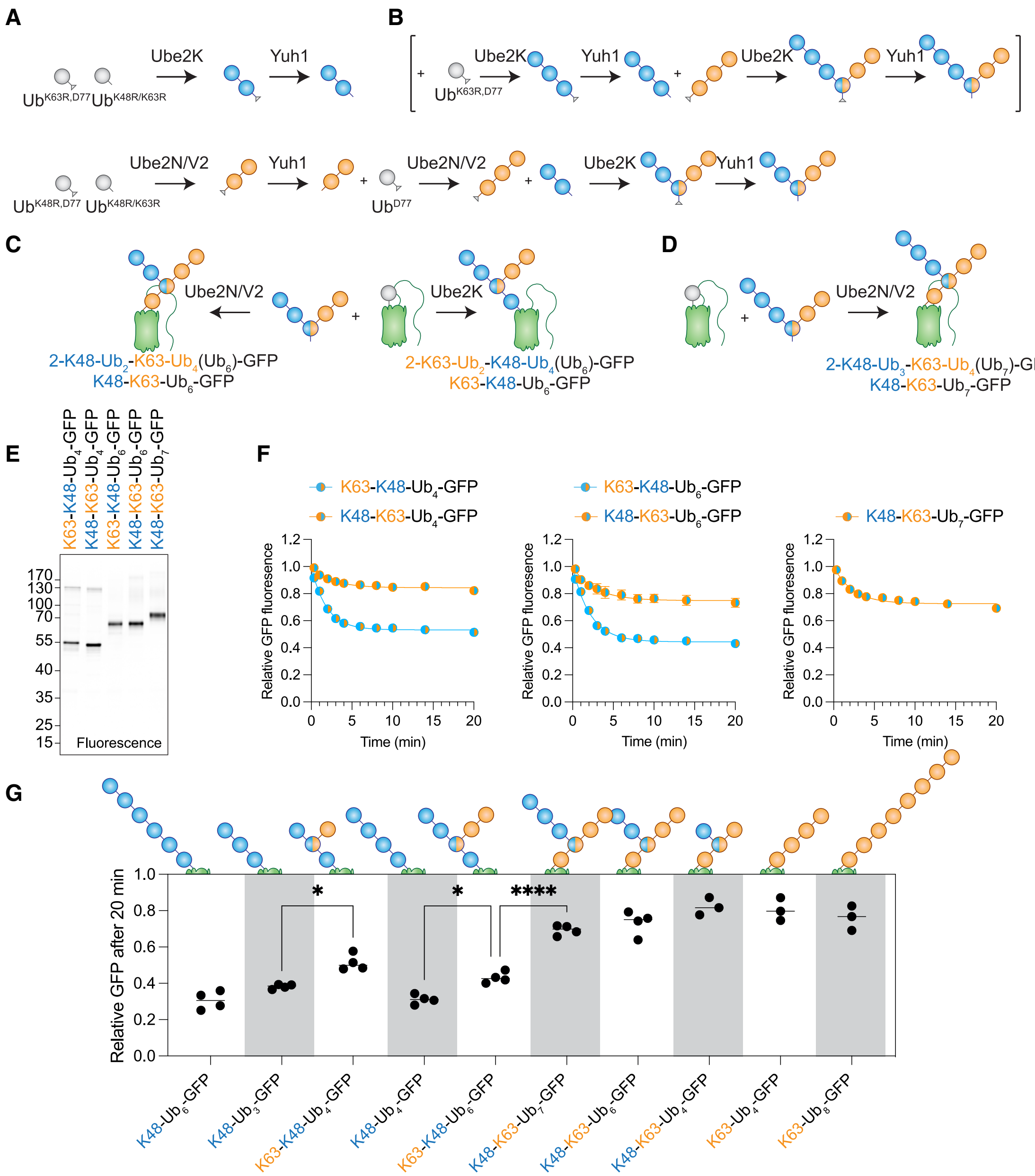

Figure 4. K48/K63-branched chains establish a degradation code inside cells

(A) Multi-step synthesis strategy for the generation of K48/K63-branched Ub_5 (1-K48-Ub_2-K63-Ub_3(Ub_5)). Nomenclature for naming of branched Ub chains by the Kulathu lab.[60]

(B) Extension of the multi-step synthesis strategy shown in (A) to generate K48/K63-Ub_6 (1-K63-Ub_2-K48-Ub_4(Ub_6)).

(C) Synthesis strategy for the conjugation of K48/K63-branched Ub_5 to Ub-GFP for the formation of K48/K63-Ub_6-GFPs (2-K48-Ub_2-K63-Ub_4(Ub_6)-GFP/K48-K63-Ub_6-GFP and 2-K63-Ub_2-K48-Ub_4(Ub_6)-GFP/K63-K48-Ub_6-GFP).

(D) Synthesis strategy for the conjugation of K48/K63-branched Ub_6 to Ub-GFP to generate 2-K48-Ub_3-K63-Ub_4(Ub_7)-GFP/K48-K63-Ub_7-GFP.

(E) In-gel fluorescence of SDS-PAGE of non-boiled K48/K63-Ub_n-GFP. Same gel stained with Coomassie is shown in Figure S4D.

(F) Relative GFP signal over time of K48/K63-$Ub_{4/6/7}$-GFP in RPE-1 cells. Data are represented as mean ± SEM of *n* independent experiments with $n = 3$ (K48-K63-Ub_4-GFP) and 4 (all other substrates).

(G) Relative GFP fluorescence after 20 min from cellular degradation kinetics shown in (F) and of K48-Ub_n-GFP (Figure 2B) and K63-Ub_n-GFP (Figure 3B). Statistics originate from ordinary one-way ANOVA, $^{*}p < 0.0332$, $^{****}p < 0.0001$.

the anaphase-promoting complex/cyclosome during cell division[19,20] or during quality control[62,63] and K29/K48 chains during proteolysis-targeting-chimera (PROTAC)-mediated degradation.[23] Although K48/K63 chains did not enhance GFP degradation, other branched Ub chains may behave differently. Given the diametrically opposite degradation phenotypes of K48-K63 and K63-K48 chains, it will be important to test if other branches also possess a functional hierarchy. This will likely depend on their stability toward DUBs and whether they form additional interfaces that increase recognition of the degradation machinery. Moreover, the potential topological arrangements of branched chains are vast, ranging from the formation of one chain on another to the formation of multiple branches or short branched chains on a substrate-anchored Ub chain. Investigating these diverse topologies will be essential for understanding their functions inside cells. With K48 chains showing high degradation capacity and velocity, it will also be crucial to understand the apparent need for branched Ub chains during degradation. UbiREAD demonstrates the power of cytosolic delivery for understanding basic biology by integrating cell biology and biochemistry. Overall, our work provides exciting insights into Ub-dependent protein degradation, deubiquitination, and the complexity of the Ub code.

Limitations of the study

Electroporation enables cytosolic delivery, but the influence of subcellular compartmentalization is not addressed by this method, for instance, excluding nuclear Ub signaling from our analysis. Information may also be encoded by the process of forming the Ub chain, which we perform outside of the cell. This would be the case for E3 ligases that not only modify their target protein but also recruit additional factors regulating degradation. We systematically compared the effect of many different Ub chains using one substrate protein, but many cellular proteins are unlike GFP. Thus, other ubiquitinated substrates with different properties need to be explored in the future.

To obtain defined ubiquitinated proteins with sufficient purity and yield, protein engineering was required, including the attachment of Ub to the GFP N terminus and K-to-R mutation in the distal Ub of homotypic and most Ubs of branched chains. In the future, chemical protein synthesis or chemical biology approaches may be used to overcome limitations in the synthesis of ubiquitinated substrates. Since studies using purified yeast proteasomes have shown that different ubiquitination sites on the same protein can influence degradation behavior,[9,64] future studies need to determine the impact of the conjugation site on intracellular protein degradation.

RESOURCE AVAILABILITY

Lead contact

Further information and requests for resources and reagents should be directed to and will be fulfilled by the lead contact, Leo Kiss (lkiss@biochem.mpg.de).

Materials availability

All unique/stable reagents generated in this study are listed in the key resources table and are available from the lead contact with a completed material transfer agreement.

Data and code availability

- Raw images (e.g., in-gel fluorescence scans of gels and Coomassie-stained gels) have been deposited at Mendeley data and are publicly available as of the day of publication. The doi is listed in the key resources table. Mass spectrometry proteomics data have been deposited and will be available at the ProteomeXchange Consortium via PRIDE[65] partner repository and are also provided at Mendeley data. The dataset identifier is listed in the key resources table.
- The paper does not report original code.
- Any additional information required to reanalyze the data reported in this paper is available from the lead contact upon request.

ACKNOWLEDGMENTS

We thank the Max Planck Institute of Biochemistry (MPIB) imaging (Martin Spitaler, Markus Oster, and Giovanni Cardone; Research Resource Identifier [RRID]: SCR_025739) and mass spectrometry facilities (Barbara Steigenberger, Anja Wehner; RRID: SCR_025745). We thank Susanne von Gronau, Shannon Smyly, Dominik Magyar, and Lara Lunglmeir for technical assistance and Nadine Renner, Jakob Farnung, Dean Clift, Bastian Bräuning, Lukas Henneberg, Laura Hehl, Arno Apli, Filiz Civril, and Gary Kleiger for discussions about the project and manuscript. L.K. was supported by an EMBO Long Term Fellowship (ALTF 627-2022), B.A.S. by the Max Planck Society, and L.C.J. by the Medical Research Council (UK; U105181010).

AUTHOR CONTRIBUTIONS

Conceptualization, L.K.; methodology, L.K.; investigation, L.K.; writing—original draft, L.K.; writing—review and editing, L.K., B.A.S., and L.C.J.; funding acquisition, L.K., B.A.S., and L.C.J.

DECLARATION OF INTERESTS

B.A.S. is a member of the scientific advisory board of Proxygen and is a co-inventor of intellectual property licensed to Cinsano. L.C.J. is a founder of TRIMTECH Therapeutics.

DECLARATION OF GENERATIVE AI AND AI-ASSISTED TECHNOLOGIES IN THE WRITING PROCESS

During the preparation of this work, the authors used Meta LLaMa 3.1 70B Instruct in order to improve the readability of their writing. After using this tool, the authors reviewed and edited content as needed and take full responsibility for the concept of the publication.

STAR★METHODS

Detailed methods are provided in the online version of this paper and include the following:

- KEY RESOURCES TABLE
- EXPERIMENTAL MODEL AND STUDY PARTICIPANT DETAILS
- METHOD DETAILS
  - Molecular Cloning
  - Degradation reporter design
  - Protein Expression & Purification
  - Synthesis of homotypic Ub chains
  - Synthesis of branched Ub chains
  - Synthesis of ubiquitinated proteins
  - Electroporation
  - UbiREAD for degradation kinetics
  - UbiREAD with in-gel fluorescence
  - Light microscopy
  - UbiCRest
  - Mass spectrometry
- QUANTIFICATION AND STATISTICAL ANALYSIS

- ADDITIONAL RESOURCES
  - Detailed protocol

SUPPLEMENTAL INFORMATION

Supplemental information can be found online at https://doi.org/10.1016/j.molcel.2025.02.021.

Received: August 21, 2024
Revised: November 13, 2024
Accepted: February 25, 2025
Published: March 24, 2025

REFERENCES

1. Komander, D., and Rape, M. (2012). The ubiquitin code. Annu. Rev. Biochem. *81*, 203–229. https://doi.org/10.1146/annurev-biochem-060310-170328.
2. Dikic, I., and Schulman, B.A. (2023). An expanded lexicon for the ubiquitin code. Nat. Rev. Mol. Cell Biol. *24*, 273–287. https://doi.org/10.1038/s41580-022-00543-1.
3. Hansen, F.M., Tanzer, M.C., Brüning, F., Bludau, I., Stafford, C., Schulman, B.A., Robles, M.S., Karayel, O., and Mann, M. (2021). Data-independent acquisition method for ubiquitinome analysis reveals regulation of circadian biology. Nat. Commun. *12*, 254. https://doi.org/10.1038/s41467-020-20509-1.
4. Crowe, S.O., Rana, A.S.J.B., Deol, K.K., Ge, Y., and Strieter, E.R. (2017). Ubiquitin Chain Enrichment Middle-Down Mass Spectrometry Enables Characterization of Branched Ubiquitin Chains in Cellulo. Anal. Chem. *89*, 4428–4434. https://doi.org/10.1021/acs.analchem.6b03675.
5. Swatek, K.N., Usher, J.L., Kueck, A.F., Gladkova, C., Mevissen, T.E.T., Pruneda, J.N., Skern, T., and Komander, D. (2019). Insights into ubiquitin chain architecture using Ub-clipping. Nature *572*, 533–537. https://doi.org/10.1038/s41586-019-1482-y.
6. Dammer, E.B., Na, C.H., Xu, P., Seyfried, N.T., Duong, D.M., Cheng, D., Gearing, M., Rees, H., Lah, J.J., Levey, A.I., et al. (2011). Polyubiquitin linkage profiles in three models of proteolytic stress suggest the etiology of Alzheimer disease. J. Biol. Chem. *286*, 10457–10465. https://doi.org/10.1074/jbc.M110.149633.
7. Thrower, J.S., Hoffman, L., Rechsteiner, M., and Pickart, C.M. (2000). Recognition of the polyubiquitin proteolytic signal. EMBO J. *19*, 94–102. https://doi.org/10.1093/emboj/19.1.94.
8. Hofmann, R.M., and Pickart, C.M. (2001). In vitro assembly and recognition of Lys-63 polyubiquitin chains. J. Biol. Chem. *276*, 27936–27943. https://doi.org/10.1074/jbc.M103378200.
9. Martinez-Fonts, K., Davis, C., Tomita, T., Elsasser, S., Nager, A.R., Shi, Y., Finley, D., and Matouschek, A. (2020). The proteasome 19S cap and its ubiquitin receptors provide a versatile recognition platform for substrates. Nat. Commun. *11*, 477. https://doi.org/10.1038/s41467-019-13906-8.
10. Martinez-Fonts, K., and Matouschek, A. (2016). A Rapid and Versatile Method for Generating Proteins with Defined Ubiquitin Chains. Biochemistry *55*, 1898–1908. https://doi.org/10.1021/acs.biochem.5b01310.
11. Bard, J.A.M., Bashore, C., Dong, K.C., and Martin, A. (2019). The 26S Proteasome Utilizes a Kinetic Gateway to Prioritize Substrate Degradation. Cell *177*, 286–298.e15. https://doi.org/10.1016/j.cell.2019.02.031.
12. Reichard, E.L., Chirico, G.G., Dewey, W.J., Nassif, N.D., Bard, K.E., Millas, N.E., and Kraut, D.A. (2016). Substrate Ubiquitination Controls the Unfolding Ability of the Proteasome. J. Biol. Chem. *291*, 18547–18561. https://doi.org/10.1074/jbc.M116.720151.
13. Saeki, Y., Kudo, T., Sone, T., Kikuchi, Y., Yokosawa, H., Toh-e, A., and Tanaka, K. (2009). Lysine 63-linked polyubiquitin chain may serve as a targeting signal for the 26S proteasome. EMBO J. *28*, 359–371. https://doi.org/10.1038/emboj.2008.305.
14. Lu, Y., Lee, B.H., King, R.W., Finley, D., and Kirschner, M.W. (2015). Substrate degradation by the proteasome: a single-molecule kinetic analysis. Science *348*, 1250834. https://doi.org/10.1126/science.1250834.
15. Hershko, A., Ciechanover, A., Heller, H., Haas, A.L., and Rose, I.A. (1980). Proposed role of ATP in protein breakdown: conjugation of protein with multiple chains of the polypeptide of ATP-dependent proteolysis. Proc. Natl. Acad. Sci. USA *77*, 1783–1786. https://doi.org/10.1073/pnas.77.4.1783.
16. Ciechanover, A., Heller, H., Elias, S., Haas, A.L., and Hershko, A. (1980). ATP-dependent conjugation of reticulocyte proteins with the polypeptide required for protein degradation. Proc. Natl. Acad. Sci. USA *77*, 1365–1368. https://doi.org/10.1073/pnas.77.3.1365.
17. Chau, V., Tobias, J.W., Bachmair, A., Marriott, D., Ecker, D.J., Gonda, D.K., and Varshavsky, A. (1989). A multiubiquitin chain is confined to specific lysine in a targeted short-lived protein. Science *243*, 1576–1583. https://doi.org/10.1126/science.2538923.
18. Spence, J., Sadis, S., Haas, A.L., and Finley, D. (1995). A ubiquitin mutant with specific defects in DNA repair and multiubiquitination. Mol. Cell. Biol. *15*, 1265–1273. https://doi.org/10.1128/MCB.15.3.1265.
19. Meyer, H.J., and Rape, M. (2014). Enhanced protein degradation by branched ubiquitin chains. Cell *157*, 910–921. https://doi.org/10.1016/j.cell.2014.03.037.
20. Yau, R.G., Doerner, K., Castellanos, E.R., Haakonsen, D.L., Werner, A., Wang, N., Yang, X.W., Martinez-Martin, N., Matsumoto, M.L., Dixit, V.M., and Rape, M. (2017). Assembly and Function of Heterotypic Ubiquitin Chains in Cell-Cycle and Protein Quality Control. Cell *171*, 918–933.e20. https://doi.org/10.1016/j.cell.2017.09.040.
21. Ohtake, F., Tsuchiya, H., Saeki, Y., and Tanaka, K. (2018). K63 ubiquitylation triggers proteasomal degradation by seeding branched ubiquitin chains. Proc. Natl. Acad. Sci. USA *115*, E1401–E1408. https://doi.org/10.1073/pnas.1716673115.
22. Akizuki, Y., Morita, M., Mori, Y., Kaiho-Soma, A., Dixit, S., Endo, A., Shimogawa, M., Hayashi, G., Naito, M., Okamoto, A., et al. (2023). cIAP1-based degraders induce degradation via branched ubiquitin architectures. Nat. Chem. Biol. *19*, 311–322. https://doi.org/10.1038/s41589-022-01178-1.
23. Kaiho-Soma, A., Akizuki, Y., Igarashi, K., Endo, A., Shoda, T., Kawase, Y., Demizu, Y., Naito, M., Saeki, Y., Tanaka, K., and Ohtake, F. (2021). TRIP12 promotes small-molecule-induced degradation through K29/K48-branched ubiquitin chains. Mol. Cell *81*, 1411–1424.e7. https://doi.org/10.1016/j.molcel.2021.01.023.
24. Kim, H.T., Kim, K.P., Lledias, F., Kisselev, A.F., Scaglione, K.M., Skowyra, D., Gygi, S.P., and Goldberg, A.L. (2007). Certain pairs of ubiquitin-conjugating enzymes (E2s) and ubiquitin-protein ligases (E3s) synthesize nondegradable forked ubiquitin chains containing all possible isopeptide linkages. J. Biol. Chem. *282*, 17375–17386. https://doi.org/10.1074/jbc.M609659200.
25. Fottner, M., Brunner, A.D., Bittl, V., Horn-Ghetko, D., Jussupow, A., Kaila, V.R.I., Bremm, A., and Lang, K. (2019). Site-specific ubiquitylation and SUMOylation using genetic-code expansion and sortase. Nat. Chem. Biol. *15*, 276–284. https://doi.org/10.1038/s41589-019-0227-4.
26. Renz, C., Asimaki, E., Meister, C., Albanèse, V., Petriukov, K., Krapoth, N.C., Wegmann, S., Wollscheid, H.P., Wong, R.P., Fulzele, A., et al. (2024). Ubiquiton-An inducible, linkage-specific polyubiquitylation tool. Mol. Cell *84*, 386–400.e11. https://doi.org/10.1016/j.molcel.2023.11.016.
27. Wegmann, S., Meister, C., Renz, C., Yakoub, G., Wollscheid, H.P., Takahashi, D.T., Mikicic, I., Beli, P., and Ulrich, H.D. (2022). Linkage reprogramming by tailor-made E3s reveals polyubiquitin chain requirements in DNA-damage bypass. Mol. Cell *82*, 1589–1602.e5. https://doi.org/10.1016/j.molcel.2022.02.016.

CellPress
OPEN ACCESS

28. Wohlever, M.L., Nager, A.R., Baker, T.A., and Sauer, R.T. (2013). Engineering fluorescent protein substrates for the AAA+ Lon protease. Protein Eng. Des. Sel. *26*, 299–305. https://doi.org/10.1093/protein/gzs105.

29. Theillet, F.X., Binolfi, A., Bekei, B., Martorana, A., Rose, H.M., Stuiver, M., Verzini, S., Lorenz, D., van Rossum, M., Goldfarb, D., and Selenko, P. (2016). Structural disorder of monomeric alpha-synuclein persists in mammalian cells. Nature *530*, 45–50. https://doi.org/10.1038/nature16531.

30. Clift, D., McEwan, W.A., Labzin, L.I., Konieczny, V., Mogessie, B., James, L.C., and Schuh, M. (2017). A Method for the Acute and Rapid Degradation of Endogenous Proteins. Cell *171*, 1692–1706.e18. https://doi.org/10.1016/j.cell.2017.10.033.

31. Alex, A., Piano, V., Polley, S., Stuiver, M., Voss, S., Ciossani, G., Overlack, K., Voss, B., Wohlgemuth, S., Petrovic, A., et al. (2019). Electroporated recombinant proteins as tools for in vivo functional complementation, imaging and chemical biology. eLife *8*, e48287. https://doi.org/10.7554/eLife.48287.

32. Ichikawa, S., Flaxman, H.A., Xu, W., Vallavoju, N., Lloyd, H.C., Wang, B., Shen, D., Pratt, M.R., and Woo, C.M. (2022). The E3 ligase adapter cereblon targets the C-terminal cyclic imide degron. Nature *610*, 775–782. https://doi.org/10.1038/s41586-022-05333-5.

33. Kiss, L., Rhinesmith, T., Luptak, J., Dickson, C.F., Weidenhausen, J., Smyly, S., Yang, J.C., Maslen, S.L., Sinning, I., Neuhaus, D., et al. (2023). Trim-Away ubiquitinates and degrades lysine-less and N-terminally acetylated substrates. Nat. Commun. *14*, 2160. https://doi.org/10.1038/s41467-023-37504-x.

34. Muhar, M.F., Farnung, J., Cernakova, M., Hofmann, R., Henneberg, L.T., Pfleiderer, M.M., Denoth-Lippuner, A., Kalčic, F., Nievergelt, A.S., Peters Al-Bayati, M., et al. (2025). C-terminal amides mark proteins for degradation via SCF-FBXO31. Nature *638*, 519–527. https://doi.org/10.1038/s41586-024-08475-w.

35. Mulder, M.P., Witting, K., Berlin, I., Pruneda, J.N., Wu, K.P., Chang, J.G., Merkx, R., Bialas, J., Groettrup, M., Vertegaal, A.C., et al. (2016). A cascading activity-based probe sequentially targets E1-E2-E3 ubiquitin enzymes. Nat Chem Biol *12*, 523–530. https://doi.org/10.1038/nchembio.2084.

36. Mandal, S., and Brik, A. (2022). Probing the cell delivery of synthetic diubiquitin chains. Chem. Commun. (Camb.) *58*, 8782–8785. https://doi.org/10.1039/d2cc02476d.

37. Mann, G., Sadhu, P., and Brik, A. (2022). Multiplexed Delivery of Synthetic (Un)Conjugatable Ubiquitin and SUMO2 Enables Simultaneous Monitoring of Their Localization and Function in Live Cells. ChemBioChem *23*, e202200122. https://doi.org/10.1002/cbic.202200122.

38. Hospenthal, M.K., Mevissen, T.E.T., and Komander, D. (2015). Deubiquitinase-based analysis of ubiquitin chain architecture using Ubiquitin Chain Restriction (UbiCRest). Nat. Protoc. *10*, 349–361. https://doi.org/10.1038/nprot.2015.018.

39. Prakash, S., Tian, L., Ratliff, K.S., Lehotzky, R.E., and Matouschek, A. (2004). An unstructured initiation site is required for efficient proteasome-mediated degradation. Nat. Struct. Mol. Biol. *11*, 830–837. https://doi.org/10.1038/nsmb814.

40. Williams, C., Dong, K.C., Arkinson, C., and Martin, A. (2023). The Ufd1 cofactor determines the linkage specificity of polyubiquitin chain engagement by the AAA+ ATPase Cdc48. Mol. Cell *83*, 759–769.e7. https://doi.org/10.1016/j.molcel.2023.01.016.

41. Olszewski, M.M., Williams, C., Dong, K.C., and Martin, A. (2019). The Cdc48 unfoldase prepares well-folded protein substrates for degradation by the 26S proteasome. Commun. Biol. *2*, 29. https://doi.org/10.1038/s42003-019-0283-z.

42. Boström, K., Wettesten, M., Borén, J., Bondjers, G., Wiklund, O., and Olofsson, S.O. (1986). Pulse-chase studies of the synthesis and intracellular transport of apolipoprotein B-100 in Hep G2 cells. J. Biol. Chem. *261*, 13800–13806.

43. Ingolia, N.T., Lareau, L.F., and Weissman, J.S. (2011). Ribosome profiling of mouse embryonic stem cells reveals the complexity and dynamics of mammalian proteomes. Cell *147*, 789–802. https://doi.org/10.1016/j.cell.2011.10.002.

44. Tsuchiya, H., Ohtake, F., Arai, N., Kaiho, A., Yasuda, S., Tanaka, K., and Saeki, Y. (2017). In Vivo Ubiquitin Linkage-type Analysis Reveals that the Cdc48-Rad23/Dsk2 Axis Contributes to K48-Linked Chain Specificity of the Proteasome. Mol. Cell *66*, 488–502.e7. https://doi.org/10.1016/j.molcel.2017.04.024.

45. Ji, Z., Li, H., Peterle, D., Paulo, J.A., Ficarro, S.B., Wales, T.E., Marto, J.A., Gygi, S.P., Engen, J.R., and Rapoport, T.A. (2022). Translocation of polyubiquitinated protein substrates by the hexameric Cdc48 ATPase. Mol. Cell *82*, 570–584.e8. https://doi.org/10.1016/j.molcel.2021.11.033.

46. Bodnar, N.O., Kim, K.H., Ji, Z., Wales, T.E., Svetlov, V., Nudler, E., Engen, J.R., Walz, T., and Rapoport, T.A. (2018). Structure of the Cdc48 ATPase with its ubiquitin-binding cofactor Ufd1-Npl4. Nat. Struct. Mol. Biol. *25*, 616–622. https://doi.org/10.1038/s41594-018-0085-x.

47. Bodnar, N.O., and Rapoport, T.A. (2017). Molecular Mechanism of Substrate Processing by the Cdc48 ATPase Complex. Cell *169*, 722–735.e9. https://doi.org/10.1016/j.cell.2017.04.020.

48. Deegan, T.D., Mukherjee, P.P., Fujisawa, R., Polo Rivera, C., and Labib, K. (2020). CMG helicase disassembly is controlled by replication fork DNA, replisome components and a ubiquitin threshold. eLife *9*, e60371. https://doi.org/10.7554/eLife.60371.

49. Twomey, E.C., Ji, Z., Wales, T.E., Bodnar, N.O., Ficarro, S.B., Marto, J.A., Engen, J.R., and Rapoport, T.A. (2019). Substrate processing by the Cdc48 ATPase complex is initiated by ubiquitin unfolding. Science *365*, eaax1033. https://doi.org/10.1126/science.aax1033.

50. Raasi, S., Orlov, I., Fleming, K.G., and Pickart, C.M. (2004). Binding of polyubiquitin chains to ubiquitin-associated (UBA) domains of HHR23A. J. Mol. Biol. *341*, 1367–1379. https://doi.org/10.1016/j.jmb.2004.06.057.

51. Tsuchiya, H., Burana, D., Ohtake, F., Arai, N., Kaiho, A., Komada, M., Tanaka, K., and Saeki, Y. (2018). Ub-ProT reveals global length and composition of protein ubiquitylation in cells. Nat. Commun. *9*, 524. https://doi.org/10.1038/s41467-018-02869-x.

52. Chen, Z.J., and Sun, L.J. (2009). Nonproteolytic functions of ubiquitin in cell signaling. Mol. Cell *33*, 275–286. https://doi.org/10.1016/j.molcel.2009.01.014.

53. Prus, G., Satpathy, S., Weinert, B.T., Narita, T., and Choudhary, C. (2024). Global, site-resolved analysis of ubiquitylation occupancy and turnover rate reveals systems properties. Cell *187*, 2875–2892.e21. https://doi.org/10.1016/j.cell.2024.03.024.

54. Du, M., Ea, C.K., Fang, Y., and Chen, Z.J. (2022). Liquid phase separation of NEMO induced by polyubiquitin chains activates NF-κB. Mol. Cell *82*, 2415–2426.e5. https://doi.org/10.1016/j.molcel.2022.03.037.

55. Zaffagnini, G., Savova, A., Danieli, A., Romanov, J., Tremel, S., Ebner, M., Peterbauer, T., Sztacho, M., Trapannone, R., Tarafder, A.K., et al. (2018). p62 filaments capture and present ubiquitinated cargos for autophagy. EMBO J. *37*, e98308. https://doi.org/10.15252/embj.201798308.

56. Sun, D., Wu, R., Zheng, J., Li, P., and Yu, L. (2018). Polyubiquitin chain-induced p62 phase separation drives autophagic cargo segregation. Cell Res. *28*, 405–415. https://doi.org/10.1038/s41422-018-0017-7.

57. Valentino, I.M., Llivicota-Guaman, J.G., Dao, T.P., Mulvey, E.O., Lehman, A.M., Galagedera, S.K.K., Mallon, E.L., Castañeda, C.A., and Kraut, D.A. (2024). Phase separation of polyubiquitinated proteins in UBQLN2 condensates controls substrate fate. Proc. Natl. Acad. Sci. USA *121*, e2405964121. https://doi.org/10.1073/pnas.2405964121.

58. Ohtake, F., Saeki, Y., Ishido, S., Kanno, J., and Tanaka, K. (2016). The K48-K63 Branched Ubiquitin Chain Regulates NF-κB Signaling. Mol. Cell *64*, 251–266. https://doi.org/10.1016/j.molcel.2016.09.014.

59. Lange, S.M., McFarland, M.R., Lamoliatte, F., Carroll, T., Krshnan, L., Perez-Rafols, A., Kwasna, D., Shen, L., Wallace, I., Cole, I., et al. (2024). VCP/p97-associated proteins are binders and debranching enzymes of

K48-K63-branched ubiquitin chains. Nat Struct Mol Biol. *31*, 1872–1887. https://doi.org/10.1038/s41594-024-01354-y.

60. Lange, S.M., McFarland, M.R., Lamoliatte, F., Kwaśna, D., Shen, L., Wallace, I., Cole, I., Armstrong, L.A., Knebel, A., Johnson, C., et al. (2023). Comprehensive approach to study branched ubiquitin chains reveals roles for K48-K63 branches in VCP/p97-related processes. Preprint at bioRxiv. https://doi.org/10.1101/2023.01.10.523363.
61. Waltho, A., Popp, O., Lenz, C., Pluska, L., Lambert, M., Dötsch, V., Mertins, P., and Sommer, T. (2024). K48- and K63-linked ubiquitin chain interactome reveals branch- and length-specific ubiquitin interactors. Life Sci. Alliance *7*, e202402740. https://doi.org/10.26508/lsa.202402740.
62. Leto, D.E., Morgens, D.W., Zhang, L., Walczak, C.P., Elias, J.E., Bassik, M.C., and Kopito, R.R. (2019). Genome-wide CRISPR Analysis Identifies Substrate-Specific Conjugation Modules in ER-Associated Degradation. Mol. Cell *73*, 377–389.e11. https://doi.org/10.1016/j.molcel.2018.11.015.
63. Samant, R.S., Livingston, C.M., Sontag, E.M., and Frydman, J. (2018). Distinct proteostasis circuits cooperate in nuclear and cytoplasmic protein quality control. Nature *563*, 407–411. https://doi.org/10.1038/s41586-018-0678-x.
64. Carroll, E.C., Greene, E.R., Martin, A., and Marqusee, S. (2020). Site-specific ubiquitination affects protein energetics and proteasomal degradation. Nat. Chem. Biol. *16*, 866–875. https://doi.org/10.1038/s41589-020-0556-3.
65. Perez-Riverol, Y., Bai, J., Bandla, C., García-Seisdedos, D., Hewapathirana, S., Kamatchinathan, S., Kundu, D.J., Prakash, A., Frericks-Zipper, A., Eisenacher, M., et al. (2022). The PRIDE database resources in 2022: a hub for mass spectrometry-based proteomics evidences. Nucleic Acids Res. *50*, D543–D552. https://doi.org/10.1093/nar/gkab1038.
66. Baek, K., Krist, D.T., Prabu, J.R., Hill, S., Klügel, M., Neumaier, L.M., von Gronau, S., Kleiger, G., and Schulman, B.A. (2020). NEDD8 nucleates a multivalent cullin-RING-UBE2D ubiquitin ligation assembly. Nature *578*, 461–466. https://doi.org/10.1038/s41586-020-2000-y.
67. Kiss, L., Zeng, J., Dickson, C.F., Mallery, D.L., Yang, J.C., McLaughlin, S.H., Boland, A., Neuhaus, D., and James, L.C. (2019). A tri-ionic anchor mechanism drives Ube2N-specific recruitment and K63-chain ubiquitination in TRIM ligases. Nat. Commun. *10*, 4502. https://doi.org/10.1038/s41467-019-12388-y.
68. Schindelin, J., Arganda-Carreras, I., Frise, E., Kaynig, V., Longair, M., Pietzsch, T., Preibisch, S., Rueden, C., Saalfeld, S., Schmid, B., et al. (2012). Fiji: an open-source platform for biological-image analysis. Nat. Methods *9*, 676–682. https://doi.org/10.1038/nmeth.2019.
69. Gibson, D.G., Young, L., Chuang, R.Y., Venter, J.C., Hutchison, C.A., 3rd, and Smith, H.O. (2009). Enzymatic assembly of DNA molecules up to several hundred kilobases. Nat. Methods *6*, 343–345. https://doi.org/10.1038/nmeth.1318.
70. Pickart, C.M., and Raasi, S. (2005). Controlled synthesis of polyubiquitin chains. Methods Enzymol. *399*, 21–36. https://doi.org/10.1016/S0076-6879(05)99002-2.
71. Michel, M.A., Elliott, P.R., Swatek, K.N., Simicek, M., Pruneda, J.N., Wagstaff, J.L., Freund, S.M.V., and Komander, D. (2015). Assembly and specific recognition of k29- and k33-linked polyubiquitin. Mol. Cell *58*, 95–109. https://doi.org/10.1016/j.molcel.2015.01.042.
72. Cox, J., and Mann, M. (2008). MaxQuant enables high peptide identification rates, individualized p.p.b.-range mass accuracies and proteome-wide protein quantification. Nat. Biotechnol. *26*, 1367–1372. https://doi.org/10.1038/nbt.1511.

STAR★METHODS

KEY RESOURCES TABLE

REAGENT or RESOURCE	SOURCE	IDENTIFIER
Bacterial and virus strains		
E.coli Rosetta	MPIB	N/A
E.coli DH5a	MPIB	N/A
E.coli BL21 Gold	MPIB	N/A
Chemicals, peptides, and recombinant proteins		
C0mplete EDTA-free protease inhibitor cocktail	Roche	Cat#05056489001
MG132	Thermo Scientific	Cat#J63250
CB-5083	MedChemExpress	Cat#HY-12861
NMS-873	Sigma-Aldrich	Cat#SML1128
TAK-243/MLN7243	MedChemExpress	Cat#HY-100487
Bafilomycin A1	Cell Signaling Technologies	Cat#54645S
MRT68921	Sigma-Aldrich	Cat#SML1644
LLOMe (H-Leu-Leu-OMe Hydrochloride)	Santa Cruz Biotechnology	Cat#sc-285992B
CCCP (Carbonyl Cyanide m-chlorophenyl hydrazone)	Selleck Chem (Absource)	Cat#S6494
Tanespimycin (17-AAG)	MedChemExpress	Cat#HY-10211-5
N-Ethylmaleimide (NEM)	Sigma-Aldrich	Cat#E3876
Tobacco Etch Virus (TEV) protease	MPIB	N/A
Critical commercial assays		
Micro BCA Protein Assay Kit	Thermo Scientific	Cat#23235
Deposited data		
Raw image data, data shown in graphs	This study	Mendeley Data: https://doi.org/10.17632/jk2vdc9srf.1
Proteomics data	This study	PRIDE: PXD060731
Experimental models: Cell lines		
hTERT RPE-1	ATCC	Cat#CRL-4000
THP-1	ATCC	Cat#TIB-202
U2OS	ATCC	Cat#HTB-96
A549	ATCC	Cat#CRM-CCL-185
HeLa	ATCC	Cat#CRM-CCL-2
293T	ATCC	Cat#CRL-3216
High-Five Insect cells	Thermo Fisher	Cat#B85502
Sf9 Insect cells	Thermo Fisher	Cat# 11496015
Recombinant DNA		
pLIB GST-TEV-UBA1	Beak et al.[66]	N/A
pGEX GST-TEV-Ube2N	This study	N/A
pOP-TG GST-TEV-Ube2V2	Kiss et al.[67]	N/A
pET His-Lipoyl-TEV-Ube2K	This study	N/A
pET His-TEV-AMSH*	This study	N/A
pET His-TEV-OTUB*	This study	N/A
pET Ub	Kiss et al.[67]	N/A
pET His-TEV-Ub-K48R	This study	N/A
pET His-TEV-Ub-K63R	This study	N/A
pET Ub-K48R-K63R	This study	N/A
pET Ub-K48R	This study	N/A

(*Continued on next page*)

Continued		
REAGENT or RESOURCE	SOURCE	IDENTIFIER
pET Ub-D77	This study	N/A
pET Ub-K48R-D77	This study	N/A
pET Ub-K63R-D77	This study	N/A
pRSF His-Yuh1	MPIB	N/A
pET cp8-sfGFP-His	This study	N/A
pET Ub-cp8-sfGFP-His	This study	N/A
Software and algorithms		
FIJI	Schindelin et al.[68]	N/A
FlowJo 10.10.0	FlowJo, LLC	N/A
Prism 10.2.3	Graphpad software	N/A
Other		
Typhoon FLA 9500	GE Healthcare	N/A
AMERSHAM ImageQuant 800	GE Healthcare	N/A
Glutathione Sepharose 4B	Cytiva	Cat#17075605
HIS-Select Nickel Affinity Gel	Sigma Aldrich	Cat#PGG11
HisTrap HP	GE Healthcare	Cat#17-5248-02
(Radioimmunoprecipitation assay) RIPA Lysis Buffer, 10x	EMD Millipore Corp.	Cat#20-188
SERVAGel TG PRiME 12%	SERVA	Cat#43286.01
Penicilin-Streptomycin	Gibco	Cat#15070063
GlutaMAX	Gibco	Cat#35050061
Fetal Bovine Serum	Gibco	Cat#10438026
Dulbecco's Modified Eagle Medium/Nutrient Mixture F-12 (DMEM/F-12), GlutaMAX Supplement	Gibco	Cat#10565018
Dulbecco's Modified Eagle Medium (DMEM)	Gibco	Cat#31966021
Roswell Park Memorial Institute 1640 (RPMI1640) media	Gibco	Cat#11875093
Sodium Pyruvate	Gibco	Cat#11360039
Trypsin-EDTA	Pan Biotech	Cat#P10-023100
Dulbecco's phosphate buffered saline (DPBS)	Gibco	Cat#14190-094
Sf-900 III Insect Cell Serum Free Medium (SFM) insect cell culture media	Gibco	Cat#12658019
Neon Transfection System	Invitrogen	Cat#10431915
Neon Transfektionssystem 10 μl-Kit	Invitrogen	Cat#10124334
Neon Transfektionssystem 100 μl-Kit	Invitrogen	Cat#10114334
Detailed UbiREAD protocol	This study	Method S1

EXPERIMENTAL MODEL AND STUDY PARTICIPANT DETAILS

RPE-1 cells (ATCC) were cultured in DMEM/F-12 + Glutamax medium supplemented with 10% Calf Serum and penicillin-streptomycin. U2OS, A549 and HeLa cells were cultured in DMEM medium supplemented with 10% calf serum, Glutamax, NaPyruvate and penicillin-streptomycin. THP-1 cells were cultured in RPMI media supplemented with 10% calf serum, Glutamax, NaPyruvate, 50 nM beta-mercaptoethanol and penicillin-streptomycin. All cells were grown at 37°C in a 5% CO_2 humidified atmosphere and regularly checked to be mycoplasma-free. The sex of RPE-1, HeLa, U2OS, 293T cells is female, the sex of THP-1 and A549 cells is male. High five insect cells were grown in Sf-900 III SFM insect cell culture media at 27 °C. High five cells are female.

Escherichia coli (E.coli) DH5a, BL21 (DE3) Gold and Rosetta were typically grown at 37 °C or 18 °C in lysogeny broth (LB), 2xTY or Terrific broth (TB) media shaking at 180 rpm.

METHOD DETAILS

Molecular Cloning

Plasmids were generated by Gibson Assembly[69] and mutations were inserted by Quick change mutagenesis. Plasmid inserts were validated by Sanger sequencing from both sides and/or full-plasmid sequencing.

CellPress
OPEN ACCESS

Degradation reporter design

The (Ub-)GFP constructs were designed based on a similar but not identical construct by the Matouschek lab.[9,10] GFP is a circular permutation of superfolder GFP (sfGFP), where strand 8 is placed at the C-terminus, strand 9 is placed at the N-terminus and strands 1 and 11 are connected (cp8-sfGFP). A methionine was added to the N-terminus as M1 of sfGFP is part of a loop after circular permutation. A yeast cytochrome b2-derived sequence where all lysines are mutated to arginine or glutamine is fused to the GFP C-terminus via a GSGS-linker followed by a 6xHis-tag for purification. Human Ub was fused directly to the N-terminus.

Protein Expression & Purification

Ube2N, Ube2V2 were expressed in *Escherichia coli* BL21 Gold, Ube2K, AMSH*, OTUB*, Ub-GFP, GFP, Ub, Yuh1 were expressed in *E. coli* Rosetta. Cells were grown at 37 °C and 220 rpm until an OD600 of ~0.7. After induction, temperature was reduced to 18 °C over night. GFPs, E2s and DUBs were induced with 0.5 mM IPTG. Ub was induced with 1 mM IPTG and expression occurred at 37 °C for 4 h. Ube2N, Ube2V2 and Uba1 were expressed as C-terminal GST fusion proteins. Ube2K as C-terminal His-Lipoyl fusion. AMSH*, OTUB* and some Ub constructs carried N-terminal His-TEV. Yuh1 carried an N-terminal His-tag and GFP constructs a C-terminal His-tag. Most Ub constructs were expressed tag-free. GST-Uba1 was expressed in *Trichoplusnia ni* High-Five insect cells by coinfection with baculoviruses prepared using SF9 insect cells.

Purification was generally performed at 4 °C. After harvest of the cultures, cells were resuspended in lysis buffer (GST lysis buffer: 50 mM Tris pH 8.0, 300 mM NaCl, 1 mM PMSF; His-lysis buffer 7.5: 50 mM HEPES pH 7.5, 300 mM NaCl, 20 mM Imidazole, 1 mM PMSF or His-lysis buffer 8: 50 mM Tris pH 8.0, 300 mM NaCl, 20 mM Imidazole, 1 mM PMSF). Lysis was performed using sonication. GST-tagged proteins were purified via glutathione Sepharose resin, equilibrated in 50 mM Tris pH 8.0, 150 mM NaCl. For E2s, tag was cleaved overnight using TEV protease and elution occurred the next day using the same buffer. In the case of GST-Uba1 fusion protein was eluted using 50 mM Tris pH 8.0, 150 mM NaCl, 10 mM reduced glutathione. TEV cleaved proteins were run over a small amount of Ni-NTA beads equilibrated in GST buffer with 20 mM Imidazole to remove TEV protease. His-tagged proteins were purified via Ni-NTA resin equilibrated in either 50 mM HEPES pH 7.5, or 50 mM Tris pH 8.0, 150 mM NaCl and 20 mM imidazole. Proteins were eluted by increasing imidazole concentration to 300 mM. His-Lipoyl-Ube2K was cleaved using TEV protease overnight during dialysis against 50 mM Tris pH 8.0, 150 mM NaCl, 20 mM imidazole and run again over Ni-NTA resin as before to remove His-Lipoyl. Finally, size-exclusion chromatography was carried out for all proteins on HiLoad 16/600 Superdex 75 or 200 prep grade columns (GE Healthcare) in 20 mM Tris pH 8.0, 150 mM NaCl for E2 enzymes and 20 mM HEPES pH 7.5, 150 mM NaCl for others.

His-TEV-Ub was purified by Ni-NTA followed by SEC as described above without TEV cleavage. In the case of untagged Ub a protocol by the Pickart lab was used.[70] After cell lysis by sonication (lysis buffer: 50 mM Tris pH 7.4, 1 mg mL^{-1} Lysozyme, 0.1 mg mL^{-1} DNAse), 0.5 % perchloric acid was added to the stirring lysate at 4 °C and the lysate was incubated for another 30 min on a stirrer at 4 °C to complete precipitation. After centrifugation at 50,000 xg for 40 min at 4 °C, the supernatant was dialyzed overnight (3,500 MWCO) against 3 L 50 mM sodium acetate pH 4.5. Ub was purified via cation-exchange chromatography using a 20 mL SP column (GE Healthcare) using a NaCl gradient (0 – 1000 mM NaCl in 50 mM NaAc pH 4.5). Finally, size exclusion chromatography was carried out on a HiLoad 16/600 Superdex 75 prep grade column (GE Healthcare) in 20 mM HEPES pH 7.5.

Synthesis of homotypic Ub chains

Homotypic Ub chains were synthesized in a highly similar fashion to the protocol of the Matouschek lab.[10] Synthesis of K48-linked Ub chains was performed in 50 mM Tris pH 8.0, 5 mM $MgCl_2$, 10 mM ATP and 0.5 mM DTT, by adding 0.5 μM E1 enzyme, 20 μM Ube2K and 7.5 mg/mL Ub and His-TEV-Ub-K48R each (876 and 692 μM, respectively). The reaction was incubated at 37 °C overnight and quenched on the next morning by addition of 5 mM DTT and incubation for 20 min at RT. Purification was performed at 4 °C. Capped Ub chains (Ub chains that contained a distal His-TEV-Ub-K48R) were purified using Ni-NTA beads equilibrated in 50 mM Tris pH 8.0, 150 mM NaCl and 20 mM imidazole. After multiple washing steps, TEV protease was added in a ratio of 1:10 (by weight) and cleavage was performed overnight. Capped but His-tag cleaved Ub chains carrying a GSGG-scar at the N-terminus of the most distal Ub were eluted the next morning using the same buffer. Cleaved Ub chains were diluted 1:10 in 50 mM NaOAc pH 4.5 and purified by size on a 6 mL ResourceS column using a gradient up to 1 M NaCl in 50 mM NaOAc pH 4.5. Peaks were neutralized using 1 mL 1 M Tris pH 8.0 per 4 mL pH 4.5 buffer and were further purified using gel filtration in 20 mM HEPES pH 7.5 on HiLoad 16/600 Superdex 75 prep grade column using a flow rate of 0.25 mL min^{-1} for optimal separation. Synthesis of capped K63 Ub chains was performed as for K48 chains, but 8 μM of Ube2N and Ube2V2 were used instead of Ube2K and 10 mg mL^{-1} Ub (1,168 μM) and 5 mg mL^{-1} His-TEV-Ub-K63R (548 μM) were added. Purification was identical to K48 chains but no SEC was required after cation exchange.

Synthesis of branched Ub chains

Formation of K48/K63-Ub_3 (1-K48-Ub-K63-Ub_2(Ub_3)) was performed in 50 mM Tris pH 8.0, 5 mM $MgCl_2$, 10 mM ATP and 0.5 mM DTT, by adding 1 μM E1, 20 μM Ube2N, 20 μM Ube2V2, 20 μM Ube2K, 1 mM Ub-K48R/K63R and 0.5 mM Ub-D77 followed by incubation at 37 °C overnight. The reaction was quenched by addition of 5 mM DTT followed by incubation for 20 min at RT. The reaction was acidified by addition of 20x volume 50 mM NaOAc pH 4.5 followed by cation exchange chromatography on 6 mL Resource S using a gradient up to 1 M NaCl. Purified 1-K48-Ub-K63-Ub_2(Ub_3)-D77 at ~0.5 mM was incubated with 2 μM Yuh1 in 50 mM HEPES pH 7.5, 1 mM EDTA for 1 h at RT. Reaction was quenched by addition of 2x 50 mM NaOAc pH 4.5 and cation exchange chromatography was repeated. A cartoon representation of this synthesis and purification is shown in Figure S4A.

Formation of K48/K63-Ub_5 (1-K48-Ub_2-K63-Ub_3(Ub_5)) was performed in the same buffer and reactions were performed at 37 °C overnight. Here, multiple reactions were performed to guarantee the exact Ub chain topology. In short, K48-Ub_2-D77 was synthesized by mixing 1 μM E1, 20 μM Ube2K, 0.5 mM Ub-K48R/K63R and 0.5 mM Ub-K63R-D77. K63-Ub_2-D77 was synthesized by mixing 1 μM E1, 20 μM Ube2N, 20 μM Ube2V2 and 0.5 mM Ub-K48R/K63R and 0.5 mM Ub-K48R-D77. Reactions were quenched and purified by cation exchange chromatography (CatEX) as described above. After deprotection with Yuh1, sample was applied to 100 μL His-NTA beads to remove Yuh1, followed by CatEX and Ub_2's were additionally purified by SEC using Superdex75 10/300 GL. K63-Ub_2 was elongated into K63-Ub_3-D77 by mixing with 1 μM E1, 20 μM Ube2N, 20 μM Ube2V2, 0.2 mM Ub-D77 and 0.2 mM K63-Ub_2. Afterwards K63-Ub_3-D77 was purified by His-NTA removal of Yuh1 followed by CatEX. To form K48/K63-Ub_5-D77, we mixed 1 μM E1, 20 μM Ube2K, 0.2 μM K48-Ub_2 and 0.2 μM K63-Ub_3-D77. This was followed by CatEX, deprotection by Yuh1, His trap and CatEX. A cartoon representation of this synthesis and purification is shown in Figure 4A.

Formation of K48/K63-Ub_6 (1-K48-Ub_3-K63-Ub_3(Ub_6)) was performed exactly as K48/K63-Ub_5 but K48-Ub_2 was elongated into K48-Ub_3, by mixing 1 μM E1, 20 μM Ube2K, 0.2 mM K48-Ub_2 and 0.2 μM Ub-D77. This was followed by CatEX, deprotection by Yuh1, Yuh1 removal by His-trap and CatEX. Then instead of using K48-Ub_2 in the final reaction, we used K48-Ub_3. A cartoon representation of this synthesis and purification is shown in Figures 4A and 4B.

Synthesis of ubiquitinated proteins

Conjugation of Ub chains to Ub-GFP was performed in 50 mM Tris pH 8.0, 5 mM $MgCl_2$, 10 mM ATP and 0.5 mM DTT, at 37 °C overnight. Reaction was performed by mixing 0.5 μM GST-E1, 10 μM Ube2K for K48 linkage or 8 μM Ube2N and 8 μM Ube2V2 for K63 linkage. Ub-GFP concentration was between 15 and 30 μM, depending on conjugation efficiency and size difference between substrate and reaction product, since large amount of Ub-GFP that are unreacted might cause trouble during purification. Generally, 15 μM Ub chain were used, unless K48-Ub_2-GFP was synthesized, where 30 μM Ub-K48R were used. The reaction was quenched by addition of 5 mM DTT and incubation for 20 min at RT. The reaction was mixed 1:1 with 50 mM Tris pH 8.0, 150 mM NaCl and applied to ~200 μL Glutathione Sepharose beads equilibrated in 50 mM Tris pH 8.0, 150 mM NaCl to remove GST-E1. Wash and Flow Through were combined, concentrated to ~0.5 mL and applied to Superdex 200 16/600. SEC was performed in 20 mM HEPES pH 7.5, 150 mM NaCl at a flow rate of 0.25 mL min^{-1}.

Electroporation

Electroporation was performed using the Neon® Transfection System (Thermo Fisher). Cells were washed with PBS and resuspended in Buffer R. For 10 μL electroporation reactions 0.8 - 1 x 10^6 cells in a volume of 11 μl were mixed with 2 μL of protein. The mixture was taken up into a 10 μl Neon® Pipette Tip, electroporated and transferred to media without antibiotics. For 100 μL electroporations, 0.5-1 x 10^7 cells in a volume of 100 μL were mixed with 18 μL of protein. Electroporation buffer E was used for 10 μL reactions and buffer E2 for 100 μL reactions, as specified by the manufacturer. Electroporation programs used were 1400 V, 20 ms, 2 pulses for RPE-1 and HeLa cells, and 1200 V, 20 ms, 2 pulses for THP-1, A549, 293T and U2OS cells. Following electroporation, cells were grown in their regular growth media without antibiotics.

UbiREAD for degradation kinetics

After electroporation, cells were added to 0.5 mL warm media with 10 % FCS but no antibiotics. Eppendorf tubes were stored with open lids in an incubator at 37 °C and 5 % CO_2. At the indicated timepoints, 40 μL of cell suspension were taken and mixed with 180 μL of ice-cold fixing buffer (5 % formaldehyde, 2 mM EDTA in PBS), typically in a 96-well U-bottom plate. In the case of longer time-courses (for GFP or Ub-GFP), cells were additionally trypsinized to receive single cells for flow cytometry. Cells were spun for ~10 min at 600 xg at 4 °C, the supernatant aspirated and the cells resuspended in 200 μL ice-cold PBS. Cell fluorescence was measured by flow cytometry on an Attune NxT Flow Cytometer. For background subtraction, either cells that were electroporated with buffer or cells that were not electroporated were used (both show identical data).

Cells were analyzed using forward and side scatterer area to assess live cells. Next, single cells were selected using front scatterer area and peak height. GFP fluorescence single cells was measured and median fluorescence intensity (MFI) exported. Only data with at least 10,000 single live cells were used and a stop gate was put at 20,000 single live cells.

Time-course data was background subtracted and fitted to a single exponential decay function: $Y = (Y_0 - Plateau) * e^{-k*t} + Plateau$, where Y is MFI, t is time (min), and k the rate of the function. Data was normalized to Y_0, to assess what happened in the dead time (20 s) of the experiment. The half-life is calculated by $Half - life = \ln(2)/k$.

For experiments in presence of drugs, cells were washed with PBS, and fresh media was added containing the indicated drug concentration 1 h before delivery. Drug was added to buffers and media including buffer R during electroporation. Drug concentration used were: 0.1 % DMSO, 1 μM TAK243, 5 μM MG132, 100 nM Bafilomycin A, 1 μM MRT68921, 1 mM LLOMe, 10 μM CCCP, 2 μM 17AAG, 5 μM NMS873 and 10 μM CB5083.

Statistics were calculated using Graphpad Prism v10 by either ordinary one-way Anova or unpaired parametric T test.

UbiREAD with in-gel fluorescence

After electroporation in 100 μL format, cells were added to 1 mL of ice-cold media and 250 μL cell suspension was taken and added to 1 mL ice-cold media. The remaining cells were put into a water bath at 37 °C and later stored with open lids in an incubator at 37 °C

and 5 % CO_2. Harvested cells were centrifuged for 30 s at 1,100 xg, supernatant was aspirated and cells were resuspended in 1 mL ice-cold PBS, centrifuged again and the pellet was snap frozen in liquid nitrogen and stored at -80 °C until further use. Given that the whole procedure takes ~2.5 min this is the first timepoint. Of note, until here, all steps were performed in ice-cold media and buffers. Later timepoints were harvested the same way, so that the final 2 min of the timepoint was in ice-cold media and buffers.

Cells were lysed in 50 μL 1xRIPA buffer, 1x Roche c0mplete® protease inhibitor and 100 mM NEM (see lysis buffer optimization in Figure S1J). Lysis was performed on ice for ~10 min and cells were vortexed roughly once every minute. Next, lysates were centrifuged for 20 min at 14,000 xg at 4°C. Lysate supernatant concentration was determined using microBCA (ThermoScientific) and 50 μL supernatant were added to 15 μL 5XSDS buffer containing beta-mercapto-ethanol. A total of 20 – 40 μg lysate was loaded onto 12 % SDS-PAGE gels and in gel fluorescence was measured on an Amersham Typhoon. As loading control, gels were stained afterwards using Coomassie stain.

Band quantification was performed using ImageJ embedded in FIJI.[68] To estimate deubiquitination rates, the band for deubiquitinated GFP and the main input band were quantified, the ratio determined and the data fit as described above. Importantly, since delivered Ub_n-GFP was not deubiquitinated, a 0 GFP value was added at time-point 0.

Light microscopy

Fixed cells (40 μL + 1 μL DAPI stain) were added to ibidi μ-Slide 15 Well 3D ibiTreat (81506). Light Microscopy was performed on a Leica Thunder inverted widefield microscope equipped with an sCMOS camera Leica DFC9000 GTC using a HC PL APO 40x/0.95 CORR air objective. Fluorescence channels was GFP (Ex 455 - 495 nm, Em 505 - 555 nm) and DAPI (Ex 375 - 435 nm, Em 450 – 490). Images were analyzed using FIJI (ImageJ).[68]

UbiCRest

UbiCRest was performed to verify the identity of Ub chains and designed based on protocols of the Komander lab.[38] Linkage-specific DUBs AMSH* (mouse Stam2-5-188 linked via a GGSSGG-linker to human AMSH-243-424) and OTUB* (human Ube2D2-C85A linked via a GGSSGGSSGG-linker to OTUB1-16-271) used were engineered by the Komander lab for use in UbiCRest, with AMSH* showing K63- and OTUB* K48-specificity.[71] Despite its high K48-specificity, minimal promiscuity was observed for OTUB*. DUBs were stored in 50 mM HEPES pH 7.5, 150 mM NaCl, 10 mM DTT. Reactions were performed in 50 mM HEPES pH 7.5, 150 mM NaCl, 5 mM DTT and contained 1 μM Ub_n-GFP and 1x DUB (100 nM AMSH* and/or 200 nM OTUB*). Reactions were mixed apart from the substrate and incubated for 10 min on RT to be sure that DUBs are reduced and active. The reactions were started by addition of the substrate and occurred at 37 °C in a thermocycler for 30 min. Reactions were stopped by addition of SDS buffer and resolved on 4 %–20 % gradient SDS-PAGE gels. Ub_n-GFP was visualized via imaging on an Amersham Typhoon.

Mass spectrometry

For total proteome measurements, $1x10^6$ RPE-1 cells were either electroporated or not and each condition was performed 3 times. Pellets were harvested after 1 h at 37 °C and 5 % CO_2, by centrifugation, followed by 2x washes with ice-cold PBS. Pellets were snap frozen in liquid N_2 and stored at -80 °C until further use. For sample preparation, the cell pellets were incubated with 100 μl of preheated SDC buffer containing 1% sodium deoxycholate (SDC, Sigma-Aldrich), 40 mM 2-chloroacetamide (CAA, Sigma-Aldrich), 10 mM tris(2-carboxyethyl)phosphine (TCEP; Thermo Fisher Scientific) and 100 mM Tris, pH 8.0. After incubation for 5 minutes at 95 °C, the samples were ultrasonicated for 10 minutes using a Bioruptor (Diogenode). Incubation for 5 minutes at 95 °C and subsequent ultrasonication was repeated. The lysates (10 μL each) were loaded in a 96 well plate for further processing with the PreOmics APP96 automation platform (PreOmics). The automated sample processing protocol involved addition of 200 μL of LYSE buffer. After incubation at 80 °C for 10 mins with shaking, the plate was cooled to 37 °C and DIGEST buffer (containing LysC and trypsin) was added. The plate was incubated at 37 °C for 1 h, and the reaction was stopped by adding 100 μL of STOP Buffer. After pipetting up and down, the content was loaded onto PreOmics cleanup cartridges (POPTips). These were washed sequentially with 200 μL wash buffer I and II. Finally, the peptides were eluted in 165 μL of ELUTE buffer. Eluted peptides were vacuum dried and was dissolved in 10μl buffer A++ (0.1% formic acid).

For LC-MS/MS data acquisition, peptides were loaded onto a 30-cm column (inner diameter: 75 microns; packed in-house with ReproSil-Pur C18-AQ 1.9-micron beads, Dr. Maisch GmbH) via the autosampler of the Thermo Easy-nLC 1200 (Thermo Fisher Scientific) at 60 °C. Using the nanoelectrospray interface, eluting peptides were directly sprayed onto the Exploris 480 mass spectrometer (Thermo Fisher Scientific). Peptides were loaded in buffer A (0.1% formic acid) and separated through the column at a flow rate of 300 nL min^{-1} by increasing percentage of buffer B (80 % acetonitrile, 0.1 % formic acid) in the following steps: increase from 5 % buffer B to 30 % buffer B over 95 mins followed by an increase to 65 % buffer B over 5 mins then 95 % over the next 5 mins. Percentage of buffer B was maintained at 95 % for another 5 mins. Over the next 5 min the percentage of B dropped down to 5 % and stayed there for 5 more min.

The mass spectrometer was operated in a data-dependent mode with survey scans from 300 to 1650 m/z (resolution of 60000 at m/z =200), and up to 15 of the top precursors were selected and fragmented using higher energy collisional dissociation (HCD with a normalized collision energy of value of 28). The MS2 spectra were recorded at a resolution of 15000 (at m/z = 200). AGC target for MS and MS2 scans were set to 3E6 and 1E5 respectively within a maximum injection time of 25 and 28 ms for MS and MS2 scans respectively. Dynamic exclusion was set to 30 s.

Raw data were processed using the MaxQuant computational platform (version 2.2.0.0)[72] with standard settings applied. Shortly, the peak list was searched against the UniProt database of human (downloaded in 2023) of with an allowed precursor mass deviation of 4.5 ppm and an allowed fragment mass deviation of 20 ppm. MaxQuant by default enables individual peptide mass tolerances, which was used in the search. Methionine oxidation and N-terminal acetylation were set as variable modifications and carbamidomethylation was set as a fixed modification. Proteins were quantified across samples using the label-free quantification algorithm in MaxQuant as label-free quantification (LFQ) intensities. The match-between-run option was enabled.

QUANTIFICATION AND STATISTICAL ANALYSIS

For the quantitative assessment of in gel fluorescence gels, these were scanned using an Amersham Typhoon and quantified using FIJI. For statistical analysis of data, ordinary one-way ANOVA with Dunnett's multiple comparison tests, or unpaired parametric two-tailed t tests were performed using Prism 10.2.3. Flow cytometry was used to quantify median fluorescence intensity (MFI) of live, single cells using FlowJo. Data fitting to a single exponential decay function was performed using Prism 10.2.3.

ADDITIONAL RESOURCES

Detailed protocol

The detailed protocol for UbiREAD is available in Method S1.

Molecular Cell, Volume *85*

Supplemental information

UbiREAD deciphers proteasomal degradation code of homotypic and branched K48 and K63 ubiquitin chains

Leo Kiss, Leo C. James, and Brenda A. Schulman

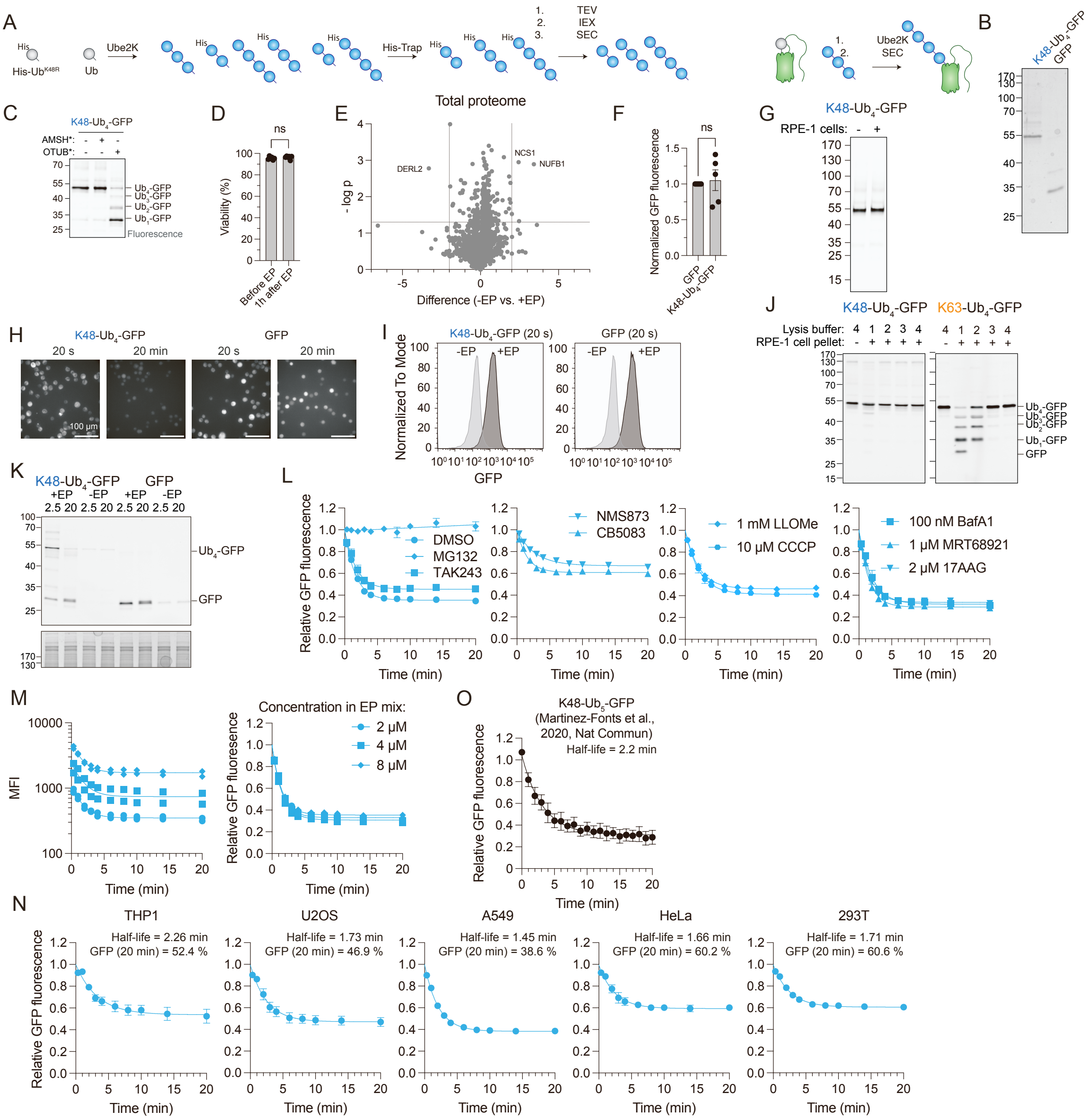

Figure S1. UbiREAD surveys intracellular ubiquitin-dependent degradation, Related to Figure 1

A Synthesis and purification strategy for capped Ub chains and their conjugation to Ub-POI.[S1] His-tagged Ub-K48R (same for K63 homotypic chains prepared in Figure 3) was incubated with Ub, E1 and E2 enzyme to generate homotypic Ub chains, where the distal Ub cannot be elongated any further. Capped and uncapped Ub chains are separated using the His-tag on the capped chains. After His-Tag cleavage of capped chains these are separated by length using cation exchange chromatography (IEX) and by size exclusion chromatography (SEC). Capped Ub chains are conjugated onto Ub-GFP using the same linkage specific E2 enzyme that was used to form the chains. Ub_n-GFP and Ub-GFP are separated by SEC.

B Coomassie stain of gel shown in Figure 1B of GFP and K48-Ub_4-GFP. Of note, additional lighter bands correspond to unfolded Ub_n-GFP.

C UbiCRest of K48-Ub_4-GFP.

D Viability determined by Trypan Blue exclusion assay before and 1 h after electroporation. Statistics originate from unpaired parametric T test (two-tailed), p=0.3343 (ns).

E Total proteome of RPE-1 cells 1 h after they were either not electroporated (-EP) or electroporated (+EP).

F K48-Ub_4-GFP delivery normalized to GFP delivery (20 s after electroporation). Statistics originate from unpaired parametric T test (two-tailed), p=0.7264 (ns).

G K48-Ub_4-GFP was incubated with RPE-1 cells exactly as in regular electroporation experiment. After 30 s, cells were not electroporated but centrifuged down and the supernatant was added to SDS buffer and run on SDS PAGE to observe Ub_n-GFP stability during cell incubation.

H Widefield microscopy showing GFP fluorescence of A549 cells.

I Flow Cytometry data testing background of non-delivered protein. K48-Ub_4-GFP or GFP were either electroporated or not electroporated and regular protocol for experiment was followed. Data show that without electroporation, no signal can be observed showing that detected GFP fluorescence is intracellular.

J Lysis buffer optimization for in gel fluorescence experiments. Non electroporated RPE-1 cell pellet was incubated with K48- or K63-Ub_4-GFP and lysis was performed in 1x RIPA buffer + c0mplete protease inhibitors (1), + 10 mM N-Ethyl-Maleimide (NEM, 2), + 100 mM NEM (3) or + 100 mM NEM + 5 mM EDTA (4). Lysis buffer 3 was chosen.

K In gel fluorescence of K48-Ub_4-GFP or GFP that were either electroporated (EP) or not in presence of RPE-1 cells to test that measured signal is intracellular rather than extracellular.

L GFP signal over time of K48-Ub_4-GFP in presence of 0.1 % DMSO, 1 µM TAK243, 5 µM MG132, 1 mM LLOMe, 10 µM CCCP, 100 nM BafA1, 1 µM MRT68921, 2 µM 17AAG, 10 µM CB5083 or 5 µM NMS873.

M Relative GFP signal over time as in Figure 1G, but here the amount K48-Ub_4-GFP was titrated and data is shown both as MFI (median fluorescence intensity) or relative GFP fluorescence. Data of the two biological replicates are shown individually.

N Relative GFP signal over time of K48-Ub_4-GFP in THP-1, U2OS, A549, HeLa and 293T cells. Error bars represent n independent experiments with n = 4 (THP-1, U2OS) or 3 (A549, HeLa, 293T).

O Biochemical single turnover assay of K48-Ub_5-GFP by yeast 26S proteasomes. Data from Source data of Martinez-Fonts et al.[S2] and fit to single exponential decay function as described for UbiREAD in methods.

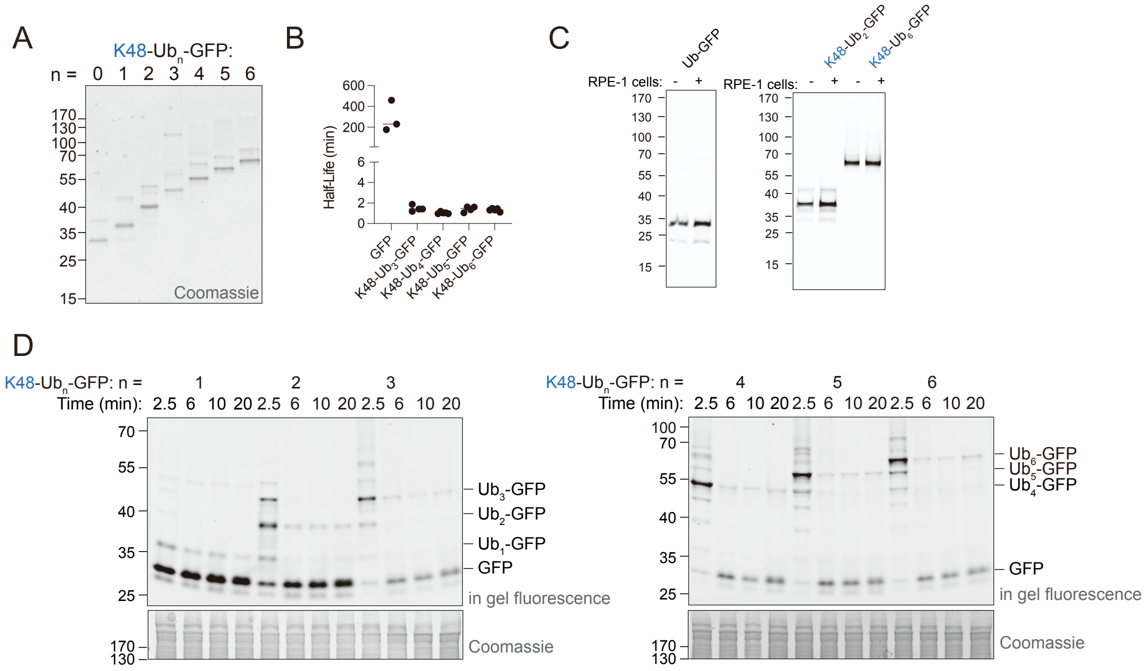

Figure S2. K48-Ub$_3$ is the minimal intracellular degradation signal, Related to Figure 2

A Coomassie stain of gel shown in Figure 2A of non-boiled K48-Ub$_n$-GFP. Of note, additional lighter bands correspond to unfolded Ub$_n$-GFP.

B Half-lives of K48-Ub$_{3/4/5/6}$-GFP and GFP of kinetics shown in Figure 2B.

C K48-Ub$_{2/6}$-GFP and Ub-GFP were incubated with RPE-1 cells exactly as in regular electroporation experiment. After 30 s, cells were not electroporated but centrifuged down and the supernatant was added to SDS buffer and run on SDS PAGE to observe Ub$_n$-GFP stability cell incubation.

D In gel fluorescence of K48-Ub$_n$-GFP delivered into RPE-1 cells showing data at 2.5, 6, 10, 20 min. Only the first and last timepoint are present in the gel in Figure 2D.

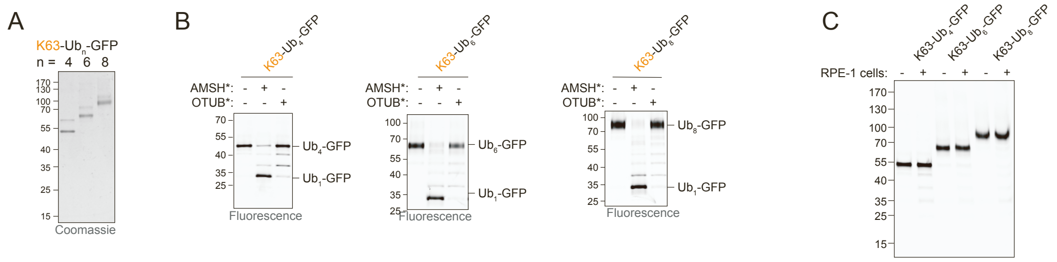

Figure S3. K63 ubiquitin chains are rapidly deubiquitinated, Related to Figure 3

A Coomassie stain of gel shown in Figure 3A of non-boiled K63-Ub_n-GFP. Of note, additional lighter bands correspond to unfolded Ub_n-GFP.

B UbiCRest of K63-$Ub_{4/6/8}$-GFP.

C K63-$Ub_{4/6/8}$-GFP were incubated with RPE-1 cells exactly as in regular electroporation experiment. After 30 s, cells were not electroporated but centrifuged down and the supernatant was added to SDS buffer and run on SDS PAGE to observe Ub_n-GFP stability during cell incubation.

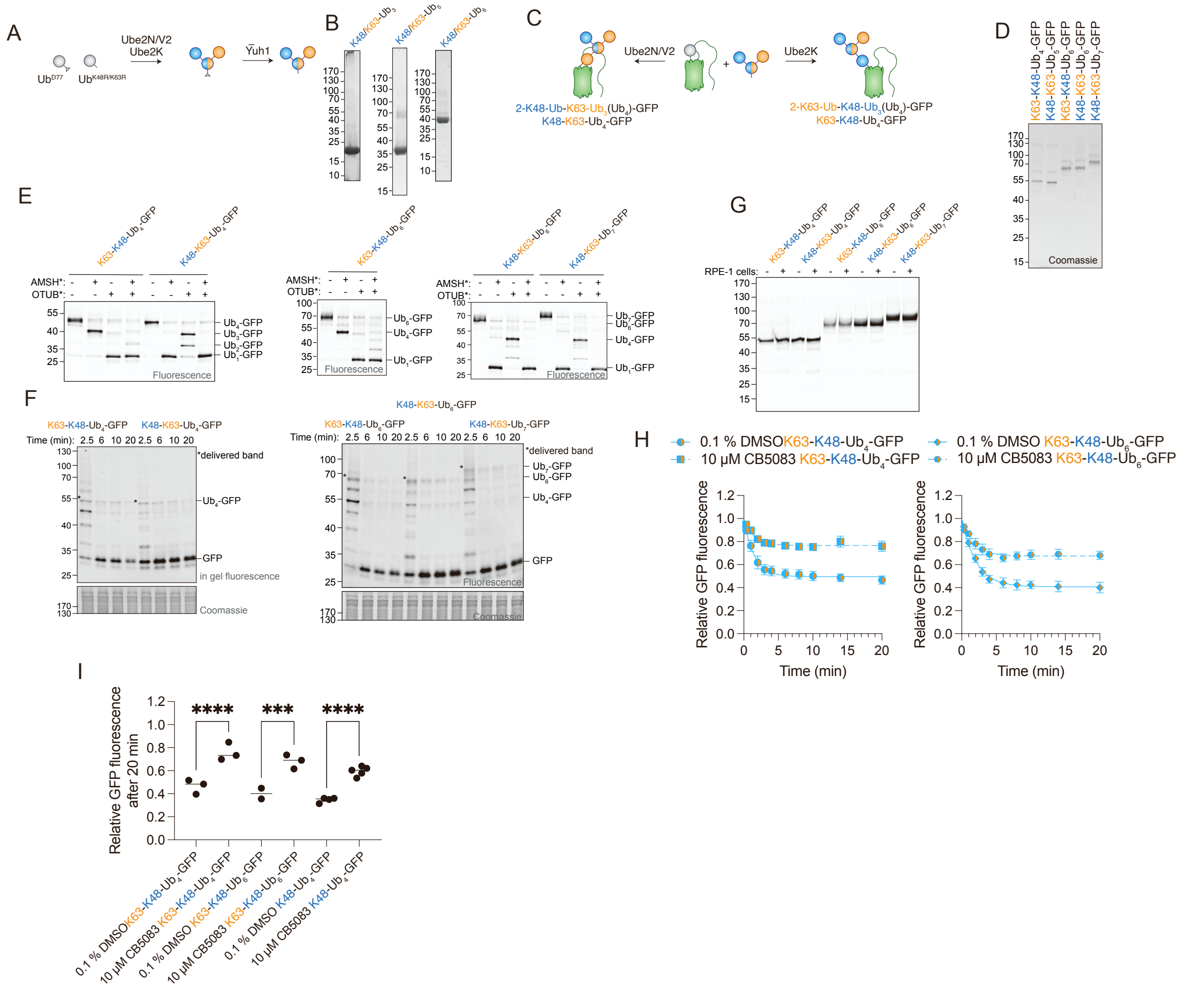

Figure S4. K48/K63-branched chains establish a degradation code inside cells, Related to Figure 4

A Synthesis strategy for K48/K63-Ub$_3$ (1-K48-Ub-K63-Ub$_2$(Ub$_3$)).

B Coomassie SDS PAGE gel of non-boiled K48/K63-Ub$_{3/5/6}$.

C Synthesis strategy for the conjugation of K48/K63-branched Ub$_3$ to Ub-GFP to generate 2-K48-Ub-K63-Ub$_3$(Ub$_4$)-GFP/K48-K63-Ub$_4$-GFP and K63-Ub-K48-Ub$_3$(Ub$_4$)-GFP/K63-K48-Ub$_4$-GFP.

D Coomassie stain of gel shown in Figure 4E of K48/K63-Ub$_{4/6/7}$-GFP. Of note, additional lighter bands correspond to unfolded Ub$_n$-GFP.

E UbiCRest of K48/K63-Ub$_{4/6/7}$-GFP.

F In gel fluorescence of K48/63-Ub$_{4/6/7}$-GFP delivered into RPE-1 cells. *(Asterisk) marks the delivered species.

G K48/K63-Ub$_{4/6/7}$-GFP were incubated with RPE-1 cells exactly as in regular electroporation experiment. After 30 s, cells were not electroporated but centrifuged down and the supernatant was added to SDS buffer and run on SDS PAGE to observe Ub$_n$-GFP stability during cell incubation.

H Relative GFP signal over time of K63-K48-Ub$_{4/6}$-GFP in presence of 0.1 % DMSO or 10 µM CB5083.

I Relative GFP fluorescence after 20 min from cellular degradation kinetics shown in H and Figure 2C and of K48-Ub_4-GFP and K63-K48-$Ub_{4/6}$-GFP. Statistics originate from ordinary one-way ANOVA, ***$p<0.0002$, ****$p<0.0001$.

Method S1. Detailed UbiREAD (Ubiquitinated Reporter Evaluation After intracellular Delivery) protocol, related to STAR Methods

UbiREAD for degradation kinetics

Electroporation was performed using the Neon® Transfection System (Thermo Fisher). Use the Neon™ Transfektions system 10 µl-Kit.

1. **General preparation:**
- Prepare 5% FACS fixative (5 % formaldehyde, 2 mM EDTA in Dulbecco's phosphate buffered saline (DPBS, 0.2 mg mL^{-1} KCl, 0.2 mg mL^{-1} KH_2PO_4, 8 mg mL^{-1} NaCl, 2.16 mg mL^{-1} Na_2HPO_4))
- Warm up regular media, antibiotic-free media, and Trypsin-EDTA mix to 37 ˚C
- Prepare 2 µL protein for electroporation in 1.5 mL sterile Eppendorf tubes
- Prepare Neon cuvette with ~3.5 mL electroporation (E) buffer
- Add 180 µL ice-cold FACS fixative into 96-well plate (or Eppendorf tube)
- Have DPBS at room temperature ready for washing cells and at 4 ˚C for changing the media of the fixed cells before flow cytometry
- Prepare 0.5 mL warm antibiotic-free media in 1.5 mL Eppendorf tubes

2. **Preparation of the cells:**
- Wash cells gently with 5 - 10 mL DPBS (RT)
- Trypsinize cells, then harvest with media and centrifuge cells (400 xg for 2 min)
- Resuspend cells in 10 mL DPBS, count and check the cellular viability (ideally >90 %)
- Take n (number of reactions + 2) x 1 x 10^6 cells and centrifuge for 2 min at 400 xg
- Aspirate supernatant and resuspend in ~1 mL DPBS and transfer cells into 1.5 mL Eppendorf tube
- Centrifuge for 2 min at 1,100 xg and aspirate supernatant
- Resuspend cells in (n + 2) x 10 µL buffer R

3. **Electroporation:**
- Add 11 µL of cells to 2 µL of protein in a 1.5 mL tube for electroporation
- Take up sample using Neon pipette and perform electroporation (1,400 V, 20 ms, 2x for RPE-1 or 1,200 V instead for 293T, U2OS, A549, THP-1)
- Add cells into 0.5 mL pre-warmed antibiotic-free media in 1.5 mL Eppendorf tube and mix
- Take out 40 µL and add into FACS fixative in ice-cold 96-well plate
- Store Eppendorf tubes with open lids in an incubator at 37 ˚C and 5 % CO_2

4. **Flow Cytometry:**
- Centrifuge 96-well plate at 600 xg for at least 5 min
- Aspirate supernatant and resuspend in 200 µL ice-cold DPBS
- Measure fluorescence at flow cytometer: use forward and side scatterer to assess live cells, then select single cells using front scatterer and peak height and measure GFP fluorescence of live single cells
- For measuring background fluorescence, either use cells that were electroporated with buffer or cells that were not electroporated (both show identical data)

5. **Data analysis**
- Export median fluorescence intensity (MFI) from data with at least 10,000 single live cells
- After background subtraction, fit data to a single exponential decay function: $Y = (Y_0 - Plateau) * e^{-k*t} + Plateau$, where Y is MFI, t is time (min), and k the rate of the function and normalize data to Y_0, to assess what happened in the dead time (20 s) of the experiment
- Estimate the half-life using $Half - life = \ln(2)/k$.

UbiREAD with in-gel fluorescence
Electroporation was performed using the Neon® Transfection System (Thermo Fisher). Use the Neon™ Transfektions system 100 µl-Kit.

Day 1:

1. **General preparation:**

- Warm up regular media, antibiotic-free media, and Trypsin-EDTA mix to 37 ˚C
- Prepare 18 µL protein for electroporation in 1.5 mL sterile Eppendorf tubes
- Prepare Neon cuvette with ~3.5 mL electroporation (E2) buffer
- Have DPBS at room temperature ready for washing cells and at 4 ˚C for changing the media of the fixed cells before flow cytometry
- Prepare ice-cold antibiotic-free media in 1.5 mL Eppendorf tubes (volume dependent on number of time-points; e.g. use 1.1 mL for 4 time-points)
- Prepare 1 mL ice-cold antibiotic-free media in 1.5 mL Eppendorf tubes for harvesting (1 / time-point)
- Prepare lysis buffer: 1x Radioimmunoprecipitation assay (RIPA) buffer (50 mM Tris-HCl pH 7.4, 150 mM NaCl, 0.25 % deoxycholic acid, 1 % NP-40, 1 mM EDTA), 1x Roche c0mplete® protease inhibitor tables, 100 mM NEM (50 µL / sample)
- Prepare 5xSDS buffer (10 g SDS, 31.2 mL Tris pH 6.8, 25 mL beta-Mercaptoethanol, 2.5 mL 2 % Bromphenol Blue, 100 mL Glycerol)
- Have a large tank of liquid nitrogen ready

2. **Preparation of the cells:**

- Wash cells gently with 5 - 10 mL DPBS (RT)
- Trypsinize cells, then harvest with media and centrifuge cells (400 xg for 2 min)
- Resuspend cells in 10 mL DPBS, count and check the cellular viability (ideally >90 %)
- Take n (number of reactions + 1) x 8 x 10^6 cells (in case of 4 time-points) and centrifuge for 2 min at 400 xg
- Aspirate supernatant and resuspend in ~1 mL DPBS and transfer cells into 1.5 mL Eppendorf tube
- Centrifuge for 2 min at 1,100 xg and aspirate supernatant
- Resuspend cells in (n + 1) x 90 µL buffer R

3. **Electroporation:**

- Add 100 µL of cells to 18 µL protein in 1.5 mL tube for electroporation
- Take up sample using Neon pipette and perform electroporation (1,400 V, 20 ms, 2x)
- Add cells into ice-cold antibiotic-free media in 1.5 mL Eppendorf tube and mix
- Take out 250 µL and add into 1 mL ice-cold media
- Centrifuge tube for 30 s at 1,100 xg, aspirate supernatant
- During centrifugation, put tube with cells into waterbath at 37 ˚C to warm them up
- Wash in 1 mL DPBS
- Centrifuge tube for 30 s at 1,100 xg, aspirate supernatant
- Put tube with cells into incubator with open lid until next harvest
- Snap-freeze pellet in liquid nitrogen
- Store pellets at -80 ˚C until further use

Day 2:

4. **Lysis and in gel fluorescence**

- Take pellets from -80 ˚C freezer and add 50 µL lysis buffer
- Perform lysis on ice for 10 min and vortex tubes every ~2 min
- Centrifuge lysates at 14,000 xg for 10 min at 4 ˚C
- Take 1 µL and add to 499 µL DPBS (for lysate concentration measurement via Micro BCA™ Protein Assay Kit)
- Take 50 µL supernatant and add to 15 µL 5x SDS buffer
- Measure lysate concentration via Micro BCA™ Protein Assay Kit according to the manufacturer description
- Load equal amounts of lysate (e.g. 20 - 40 µg) onto SDS-PAGE and run gel
- Measure in gel fluorescence on a Typhoon FLA 9500

- Afterwards, stain gel with Coomassie as loading control

5. **Data analysis**

- Band qualtification can be performed using ImageJ in FIJI
- Estimate deubiquitination rates by quantifying the deubiquitinated GFP and the main input band and fit data as described above. A value of 0 can be added for timepoint 0, since the delivered Ub_n-GFP was not deubiquitinated before delivery.

General UbiREAD Quality Control:

- Cell viability is key to a successful experiment. Check viability during counting and keep a few cells to check viability 1 h post electroporation (see data for RPE-1 cells in Figure S1D). Viability should ideally be >90 % before and after the experiment! Be particularly careful when using UbiREAD in presence of drugs as these drugs may have severe consequences for cellular health.
- Every cell line behaves differently and must be optimized and tested in electroporation. If cellular viability is low after the experiment, optimize delivery.
- Check that your protein does not get modified before electroporation for instance by incubating cells with protein of interest for the time it takes until electroporation and check the protein via SDS-PAGE (e.g. done in Figure S1G, S2C, S3C or S4G).
- Purity and homogeneity of the electroporated proteins are key for successful UbiREAD.

Supplemental References

1. Martinez-Fonts, K. and A. Matouschek, *A Rapid and Versatile Method for Generating Proteins with Defined Ubiquitin Chains.* Biochemistry, 2016. **55**(12): p. 1898-908.
2. Martinez-Fonts, K., et al., *The proteasome 19S cap and its ubiquitin receptors provide a versatile recognition platform for substrates.* Nat Commun, 2020. **11**(1): p. 477.
